# Predicting Potential PRRSV-2 Variant Emergence through Phylogenetic Inference

**DOI:** 10.1155/2024/7945955

**Published:** 2024-02-05

**Authors:** Nakarin Pamornchainavakul, Mariana Kikuti, Igor A. D. Paploski, Cesar A. Corzo, Kimberly VanderWaal

**Affiliations:** College of Veterinary Medicine, University of Minnesota, St. Paul, MN, USA

## Abstract

Porcine reproductive and respiratory syndrome (PRRS) is a significant pig disease causing substantial annual losses exceeding half a billion dollars to the United States pork industry. The cocirculation and emergence of genetically distinct PRRSV-2 viruses hinder PRRS control, especially vaccine development. Similar to other viral infections like seasonal flu and SARS-CoV-2, predictive tools for identifying potential emerging viral variants may prospectively aid in preemptive disease mitigation. However, such predictions have not been made for PRRSV-2, despite the abundance of relevant data. In this study, we analyzed a decade's worth of virus ORF5 sequences (*n* = 20,700) and corresponding metadata to identify phylogenetic-based early indicators for short-term (12 months) and long-term (24 months) variant emergence. Our analysis focuses on PRRSV-2 Lineage 1, which was the predominant lineage within the U.S. during this period. We evaluated population expansion, spatial distribution, and genetic diversity as key success metrics for variant emergence. Our findings indicate that successful variants were best characterized as those that underwent population expansion alongside widespread geographical spread but had limited genetic diversification. Conditional logistic regression revealed the local branching index as the sole informative indicator for predicting population expansion (balanced accuracy (BA) = 0.75), while ancestral branch length was strongly linked to future genetic diversity (BA = 0.79). Predicting spatial dispersion relied on the branch length and putative antigenic difference (BA = 0.67), but their causal relationships remain unclear. Although the predictive models effectively captured most emerging variants (sensitivity = 0.58–0.81), they exhibited relatively low positive predictive value (PPV = 0.09–0.16). This initial step in PRRSV-2 prediction is a crucial step for more precise prevention strategies against PRRS in the future.

## 1. Introduction

Infectious disease emergence and reemergence have posed significant challenges to human well-being over the centuries. Despite advancements in technology, the ability to preemptively prepare for such unexpected events remains limited unless there is a high degree of emergence predictability. These threats extend beyond human and zoonotic diseases that directly impact human health; they also include livestock diseases that undermine food security. Over 30 years ago, the United States witnessed the emergence of a viral disease known as porcine reproductive and respiratory syndrome virus (PRRSV) [1], which causes substantial productivity losses in commercial pig farming. Today, PRRSV is endemic in swine herds worldwide [2] and remains a significant concern due to its enormous economic consequences [3–6]. Genetic variants involved in contemporary outbreaks are distinct from the early virus [7], reflecting the rapid mutation rate of the virus [8]. Some of these variants have been associated with distinct virulence or epidemic characteristics, presenting atypical clinical manifestations [9] or increased disease spreadability [10].

In the United States, *Betaarterivirus suid 2*, also known as PRRSV-2 [11], is the predominant viral species responsible for most PRRS outbreaks [12]. The PRRSV-2 viral population is characterized by cocirculation and turnover of distinct genetic variants, and the routine emergence and epidemic-like spread of novel variants across space and time [7, 13]. To date, classification of PRRSV-2 genetic variants has primarily relied on analyzing the variation in the open reading frame 5 (ORF5) gene [7, 12, 14–16], which encodes the viral glycoprotein 5 (GP5). GP5 possesses major antigenic determinants [17] that may have evolved under immune selection pressure [8, 12]. The genetic relationship between viral clades, as demonstrated through ORF5 phylogenies, generally reflects overall genome-level relationships, particularly in cases where recombination is absent within a given set of samples [18, 19]. Over time, the operational taxonomic unit (OTU) of ORF5 has undergone revisions in its classification methodology, transitioning from restriction fragment length polymorphism patterns [16] to lineage and sublineage classification based on phylogenetic analysis [7, 12, 15]. Sublineages constitute the smallest phylogeny-based OTU, with typically less than 8.5% nucleotide dissimilarity within the group [7, 12], and are made up of finer scale genetic clades, here referred to as “variants,” that potentially exhibit heterogeneous virulence or epidemiological impacts [10, 19].

In addition to implementing biosecurity measures, vaccination plays a crucial role in mitigating clinical PRRS outbreaks on farms that have tested positive for the virus [20, 21]. While the precise mechanisms of immunity against PRRSV, particularly regarding neutralizing antibodies, have yet to be fully understood [22, 23], the genetic diversity within epitopes found on GP5 is recognized as a factor influencing immunological cross-protection. Various immune evasion mechanisms have been proposed, including epitope masking through N-glycosylation, the presence of immunodominant non-neutralizing (decoy) epitopes, and the existence of additional epitopes conferring homologous but not heterologous neutralization on GP5 [17, 24, 25]. These mechanisms are presumably associated with the variable efficacy of vaccines. Consequently, in the past decade, there has been a growing trend for selecting virus strains for immunization that are either homologous or genetically more similar to field strains. Examples include the use of field viruses as autogenous inoculums to homogenize immunity within a herd (so-called live virus inoculation), the development of new commercial vaccines that are based on the currently most prevalent phylogenetic lineages [26, 27], and the use of killed vaccines matched to the amino acid sequence of particular epitopes [28]. However, the effectiveness of using a “homologous” vaccine to confer optimal protection remains a topic of debate [22, 23, 29–32], but one key challenge for such approaches is the continual emergence of new genetic variants [33, 34].

Efforts to predict viral strain emergence have been successfully developed for certain human contagious diseases, with the aim of minimizing future outbreaks through informed vaccine strain selection. One pioneering example is the prediction of human seasonal influenza, where the fitness of different genetic variants is inferred from the branching patterns of each node on a phylogenetic tree—a metric known as the local branching index (LBI) [35]. Subsequent advancements have enhanced short-term prediction accuracy by incorporating LBI with tree shape and epitope features [36]. More recently, it has become possible to predict the emergence of SARS-CoV-2 lineages by evaluating key amino acid substitutions and spatiotemporal prevalence data from millions of genomes, without the need for complete phylogenetic tree reconstruction [37]. Surprisingly, such informative prediction techniques have not been explored for PRRSV-2, despite the continuous generation of large amounts of genetic data and corresponding metadata through ongoing monitoring and surveillance. In this study, we leveraged a decade's worth of PRRSV-2 ORF5 sequences from one of the largest swine disease monitoring databases in the U.S. Our objective was to systematically classify PRRSV-2 variants, assess their epidemiologic success over time with respect to population growth, geographic expansion, and genetic diversification, and develop predictive models that can be used to estimate a variant's future emergence potential. By identifying variants of interest at a given point of time, our proposed model offers a proactive approach and provides an additional tool for achieving more precise PRRS control.

## 2. Materials and Methods

### 2.1. Data Collection

PRRSV-2 ORF5 sequences collected from January 1, 2010 to June 30, 2021 were obtained from the Morrison Swine Health Monitoring Project (MSHMP), which is an ongoing monitoring program that archives, analyzes, and reports data related to major swine diseases. MSHMP monitors over 50% of the U.S. sow population and curates all PRRSV ORF5 sequences generated by MSHMP participants. Sequences are obtained directly from participants or from the main veterinary diagnostic laboratories where participants typically submit their diagnostic samples (University of Minnesota, Iowa State University, South Dakota State University, and Kansas State University). In U.S. swine production systems, ORF5 sequences are usually generated for almost all outbreaks on breeding farms within the system. The lineage or sublineage of each sequence is determined based on its nucleotide distance from reference sequences [7, 12]. Lineage 1 (L1) has been a predominant group (>60% of identified sequences) of PRRSV-2 circulating in the U.S. during the recent decade [7, 12, 13]. The second and third most common groups (<40%), namely L5 and L8, are almost entirely vaccine-associated [12, 38–40] and do not represent the natural evolutionary dynamics of PRRSV-2. Hence, we used only L1 ORF5 sequences for the analysis of PRRSV-2 genetic variants. For this study, 20,700 complete length (603 nucleotides) L1 ORF5 sequences were compiled and then aligned using the local alignment method in MAFFT v.7.310 [41]. All sequences had sampling date information and most sequences had corresponding spatial metadata including the U.S. state (74.8% of all sequences) and county (66.2% of all sequences) (Figure S1).

### 2.2. Phylogenetic Reconstruction and Variant Assignment

**Figure 1:**
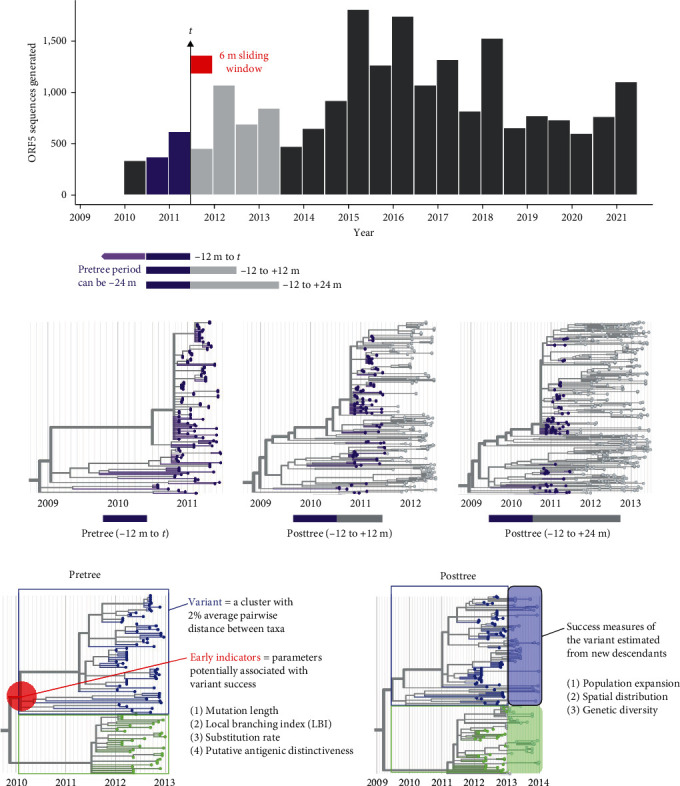
Conceptual framework of data generation for systematic predictive modeling. (a) Temporal distribution of PRRSV-2 L1 ORF5 sequences used in this study. As an example, observation time (*t*) is shown in July 2011 (vertical arrow) with its corresponding pretree (purple bars) and posttree (purple and gray bars) periods. The pretree and posttree were built for each *t* set as every six months (red bar) from 2011 to 2020. (b) Example pre- and post-timed phylogenetic trees inferred from sequencing data. Tips in purple show sequences from the pretree that are present in both posttrees. (c) Information computed in an example pretree, including designated variants (colored rectangle frames) and early indicators (red circle shows the ancestral node of the blue variant). (d) Success measures (colored oblong shape) are calculated from variants' new descendants in the posttree.

Our goal was to identify early phylogenetic indicators that were predictive of a genetic variant's future epidemiological success. Therefore, we approached this analysis by defining a series of sliding windows (Figure 1(a)) over which to quantify early indicators, and then correlate these indicators to the variant's future success in a follow-up period of time. The ORF5 alignment was used to reconstruct retrospective phylogenies across different windows of time. For each observation time (*t*), set as every 6 months starting from 1 January 2011 to 1 July 2020, we built two sets of “pretrees” (time-scaled phylogenetic trees of sequences collected within the previous 12 or 24 months before time *t*) and four sets of “posttrees” (time-scaled trees created from the same sets of sequences in the pretrees plus sequences collected within the following 12 or 24 months after time *t*) (Figures 1(a) and 1(b)). Some trees at the beginning and the end of the study period could not be built due to truncation of sequences 24 months before or after time *t*. We chose a 6-month sliding window and a 12- or 24-month observation period by considering the frequency of data sharing with the database, the yearly seasonal pattern of PRRS incidence [42, 43], and the number of sequences utilized to reconstruct a tree.

From resampled alignments generated by PHYLIP's Seqboot v.3.69, each tree was initially built by FastTree v.2.1.10 [44] using the maximum likelihood (ML) method, the GTR + CAT substitution model (generalized time-reversible with each site's rate approximation), and 100 bootstrap replicates. The ML tree bootstrap clade supports were then converted into the transfer bootstrap expectation using BOOSTER v.0.1.1 [45], as transfer bootstraps typically yield better results for phylogenetic analyses with large datasets and rapidly evolving viruses [45]. We defined PRRSV-2 variants based on the patristic distance, i.e., the sum of the shortest branch length connecting two taxa on the tree. The “Avg Clade” method of TreeCluster v.1.0.3 [46] was applied to each ML tree, which classified sequences into variants where a variant was defined as a monophyletic clade with an average pairwise patristic distance of <2% regardless of the clade support (Figure 1(c)). Using TreeTime v.0.9.2 [47], branch lengths in each ML tree were reestimated to generate two time-scaled phylogenetic trees, one tree using the default strict molecular clock model with highly diverging tips pruned and the other tree using the uncorrelated relaxed clock model without tree pruning. Highly diverging tips were tips for which residuals exceed four interquartile distances of the residual distribution in the least-square root-to-tip distance versus sampling date regression [47].

### 2.3. Early Indicators

Early indicators, which were considered as potential parameters in the predictive model, were either retrieved or calculated from the set of pretrees (Figure 1(c)). First, we located the most recent common ancestor of each variant on the tree (i.e., variant's ancestral node and branch) using Biopython v.1.81′s Bio.Phylo toolkit [48] in Python [49]. Thereafter, we calculated four key categories of parameters related to the variant's ancestor, including ancestral branch length, LBI, nucleotide substitution rate, and putative antigenic distinctiveness from contemporary most-prevalent variants.

The ancestral branch length is a length of branch from the ancestral node to the closest deeper node in the original ML tree and thus provides a metric of genetic divergence from other sequences in the tree. LBI is the sum of the tree length in each node's neighborhood, exponentially weighted by distance from the focal node [35]. Using Nextstrain's “augur lbi” command [35, 50], the LBI of each variants' ancestral node (ancestral LBI) was computed from the strict clock time-scaled tree with the tau (*τ*) parameter, which controls the size of neighborhood measured in units of the average pairwise distance in the samples [35], equal to 0.0625 times the average pairwise patristic distance of each particular tree, as recommended by Neher et al. [35]. Average pairwise patristic distance was calculated by “cophenetic.phylo” function in R's ape v.5.6.2 [51, 52]. Nucleotide substitution rates for each variants' ancestral branch (ancestral rate) were extracted from the relaxed clock time-scaled trees. We also averaged the substitution rates across all branches within a variant's clade (average clade rate).

Lastly, putative antigenic distinctiveness of each variant was measured in two ways based on the variant's ancestral GP5 sequence (translated ORF5 amino acid sequence), with the hypothesis that variant's whose sequences differ in antigenically relevant ways from the most prevalent variants at the time may be better able to escape population immunity present against those more prevalent variants. A variant's ancestral sequence was inferred as part of the relaxed clock time-scaled tree building using the “ancestral” function in TreeTime v.0.9.2 [47]. Putative antigenic distinctiveness was measured as (1) ancestral amino acid distance—a pairwise amino acid distance (“dist.aa” function in R's ape v.5.6.2) [51, 52] between the ancestral GP5 to the consensus GP5 from all samples collected in the same calendar year and (2) ancestral N-glycosylation pattern similarity—Jaccard similarity between potential N-glycosylation sites (positions having N-X-S/T sequons) [53] on the ancestral GP5 and the most frequent N-glycosylation pattern found in all samples of the recent calendar year. The Jaccard index ranges between 0 and 1, with lower values indicating fewer N-glycosylation sites in common and putatively greater antigenic dissimilarity. In total, these six parameters were considered as candidate early indicators.

### 2.4. Measures of Success

For any given timepoint, the pre- and post-trees constituted separate phylogenetic reconstructions, so the first step of measuring success in the posttree was to identify the clade that corresponded to variants identified on the pretree. Because phylogenetic construction is an imperfect best-estimate of true underlying evolutionary relationships, topological differences between the pre- and post-tree meant that not all variant's present in the pretree were readily identifiable as monophyletic clades in the posttree. We considered a pre- and post-tree variant to be matched if their members (i.e., the sequences present in both the pre- and post-tree analyses) were highly overlapping (>75% Jaccard similarity, indicating that 75% of sequence pairs belonged to the same variant in both the pre- and post-tree analyses).

Success of a variant was estimated from the new descendants of a variant in the posttree and was characterized across three aspects—population expansion, spatial distribution, and genetic diversity (Figure 1(d)). We quantified population expansion of each variant by computing the absolute and relative increases in number of taxa from the pre- to post-tree.

Spatial distribution of the variant was also estimated as the absolute and relative increases in number of states, and number of counties, in which the variant was detected. Additionally, pairwise geographical distance between county centroids were calculated between sequences belonging to the same variant. The maximum pairwise distance (as well as the 95^th^ percentile to mitigate the effect of outliers) was extracted to approximate the geographic range of a variant in each pre- and post-tree. To measure changes in geographic extent, the absolute and relative increases of pairwise county distance (based on either the maximum or 95^th^ percentile) were calculated for each posttree variant compared to its geographic extent based on the original members from the pretree.

Genetic diversity was measured as pairwise nucleotide distance (“dist.dna” function with K80 evolutionary model in R's ape v.5.6.2) [51, 52] among all members of a variant, and the 95^th^ percentile was used as the representative nucleotide distance of the variant (e.g., 95% of sequences belonging to a variant have a nucleotide distance of less that *x* distance). Then, the absolute and relative increases in nucleotide distance were calculated between the pre- and post-tree. In total, 12 features were considered as potential measures of variant success.

### 2.5. Predictive Modeling

For each of four scenarios (12 or 24 months before and after *t*), a matrix of Spearman's correlation coefficients (*ρ*) was computed and visualized between all six early indicator candidates, using “ggpairs” function from the R's GGally v.2.2.0 and ggplot2 v.3.4.3 packages [54, 55], to assess collinearity; multivariable models can be severely impacted at collinearity of |*ρ*| > 0.7 [56]. All possible sets of noncollinear candidates were used as predictor variables. Given that successful variants appeared to be rare (early data exploration showed that the distribution of success metrics was highly right skewed), and because the numerical range of success metrics likely varied depending on the size of phylogenetic tree at different periods of time, a matched case-control study was applied using the observation time (*t*) of each scenario as a matched set (stratum). For each of 12 measures of success, variants whose success measure fell in the top 95^th^ percentile were classified as a successful variants or “cases,” whereas variants in the lower 75^th^ percentile were classified as nonsuccessful variants or “controls.” Three controls were randomly selected from the same pretree for every case.

Using “clogit” function in the survival package v.3.5.0 in R [52, 57, 58], we fitted conditional logistic regression models on the training dataset using the first 8 years (2011–2018) or approximately 80% of the data. Cases and controls from the last 2 years (2019–2020) of the data were used as a test set to validate the predictive model performance. To perform prediction on the test set, as described elsewhere, we first derived the average threshold value for each predictor that minimized the misclassification rate in the training dataset, and these values were used to generate predictions for the test dataset [59].

Amongst choices of models that differ by set of predictors (early indicators), response variables (measure of success), and scenario (length of time periods considered), only the models from the training set that had *p*-values < 0.05 for likelihood ratio tests were kept for further assessment, indicating that these models performed significantly better than a null model. For each aspect (population growth, geographic extent, and genetic diversification) and each follow-up period (short- versus long-term success (12 vs. 24 months)), we selected the measure of success that has the highest performance, as measured by mean concordance (i.e., analogous to area under the ROC curve (AUC) for binary responses [60]), based on the model fitted on the training set and the highest mean balanced accuracy based on the prediction on the test set. Then, we selected the *n*-month pretree model that maximized concordance and balanced accuracy for each *n*-month posttree and selected measure of success.

Coefficients, odds ratio, and *p*-values of each predictor in the final models, and the model performance metrics, comprising sensitivity, specificity, positive predictive value (PPV), negative predictive value (NPV), F1-score, and balanced accuracy, were calculated from a confusion matrix derived from the test set predictions were reported. Furthermore, we applied these final models to predict the success of all observed variants (the full dataset) at each timepoint, aiming to evaluate the predictive performance of the data beyond the scope of the matched case-control design.

## 3. Results

A total of 74 unique sets of time-scaled phylogenetic trees with a median size of 4,247.5 (IQR = 2,688–6,712.75) taxa were reconstructed to obtain the pretrees and the posttrees at each 6-month window observation time (*t*) throughout 2011–2020 for all four scenarios (12 or 24 months before and after *t*). Classified by 2% average pairwise patristic distance, the median number of variants per tree was 151 (IQR = 96–204), the median size of a variant was 12 (IQR = 5–30) taxa per variant, and the median bootstrap clade support of variant in all trees was 84% (IQR = 63–97) (Figures S2 and S3). Only 58% of all the pretree variants could be matched (>75% Jaccard similarity between variants' members) with a posttree variant. The number of matched variants varied from 53% to 63% of the total pretree variants for each scenario (Figure S4). According to Welch two-sample *t*-test, the bootstrap clade support of pretree variants with posttree matches (mean = 85 (IQR = 80–100)%] was significantly higher (*p* < 0.001) than that of the unmatched variants (mean = 68 (IQR = 51–89)%) (Figure S5). An average of 11.7%, 24.8%, 5.3%, and 13.9% of the total posttree variants were new variants (no tips derived from the pretree period) for the 12-*t*−12, 12-*t*−24, 24-*t*−12, and 24-*t*−24 scenarios, respectively (Figure S6).

**Figure 2:**
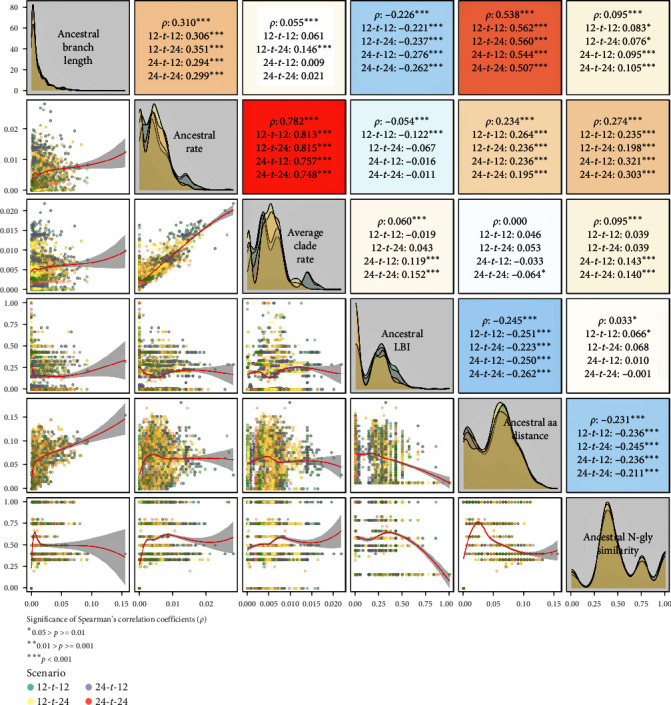
Matrix of Spearman's correlation coefficients (*ρ*) between all candidate early indicators for the overall data and each prediction scenario data with background color corresponding to the strength of correlation from 1 (red) to −1 (blue) (upper panel), their data density plots (diagonal), and bivariate scatterplots colored by the scenario with LOESS curves fitted (red line) and associated 95% confidence intervals (gray polygon) (lower panel).

The candidate early indicators of variant success were obtained from the pretree variants' ancestral nodes (branch length, LBI, substitution rate, amino acid distance, and N-glycosylation similarity) and the whole variant clade (average clade rate). LBI was the only parameter that could not be computed for all ancestral nodes due to excessive branch length (higher than four interquartile distances from the clock model regression) in several time-scaled trees. We thus computed LBI only from the time-scaled trees for which the problematic branches were pruned. This resulted in a small proportion of variants (1.1% of all 4,323 matched variants; Figure S7) sharing the same ancestral LBI because their ancestral nodes were collapsed together during the tree pruning step. Amongst all six candidate indicators, severe collinearity (|*ρ*| > 0.7) was only detected between ancestral rate and average clade rate in the overall data and in every scenario (Figure 2 and Figures S8–S11). Therefore, two models were separately fitted for each measure of success (response) from the remaining four candidate predictors plus either ancestral rate or average clade rate.

**Figure 3:**
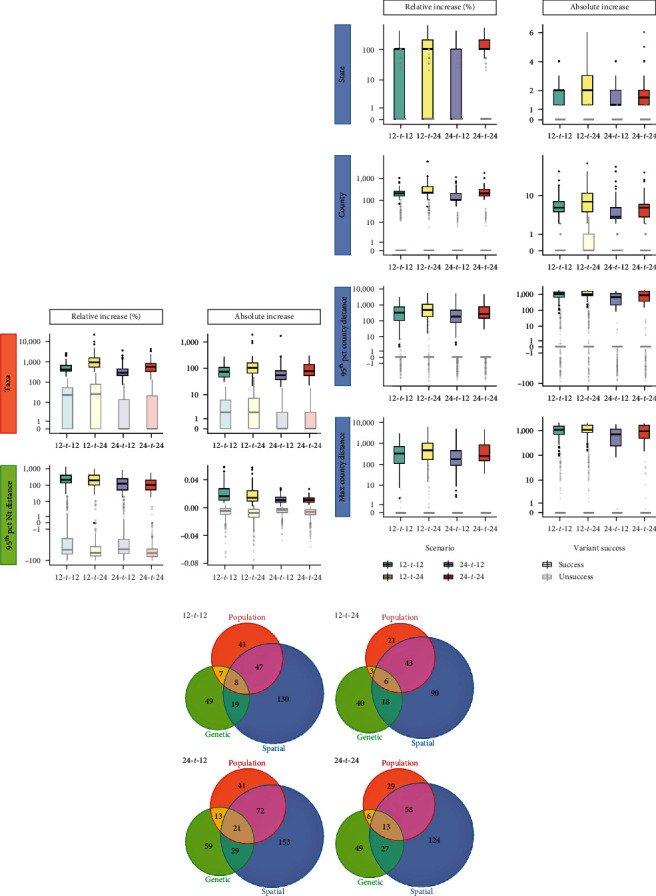
Aspects of success, success measures, and distribution of values for success vs. unsuccess of each measure. (a) Distribution of success metrics for population expansion (orange) and genetic diversity (green). (b) Distribution of success metrics for spatial distribution (blue). (c) Venn diagrams tabulating the number of variants that achieved success in one or more of the population, geographic, or genetic diversification aspects (not including success in relative increase in number of states).

Three aspects of variant success, comprising population expansion, spatial distribution, and genetic diversification, were calculated as absolute or relative increases when comparing between the matched post- and pre-tree variants. Although we did control for impact of tree size or timespan on measures of success (e.g., time periods with greater sequencing effort could influence how many additional taxa a variant could increase in the posttree) by using a matched case-control design, the numerical distributions of success metrics were relatively consistent through time and across different scenarios. For population expansion, the successful posttree variants were typically at least twice the size of the original pretree variant (median relative increase in number of taxa = 400% (IQR = 283.3–804.7)) or had at least 20 more taxa than the pretree variant (median absolute increase in number of taxa = 70 (IQR = 44.5–112.5)) (Figure 3(a) and Figure S12). For genetic diversification, successful variants increased in their genetic diversity from the pre- to post-tree by the median distance of 0.01 (IQR = 0.008–0.018) or one site per 100 nucleotides, while the diversity of nonsuccessful variants decreased by the median of 0.004 (IQR = 0.002–0.009) or 0.4 site per 100 nucleotides (Figure 3(a) and Figure S13). Geographic expansion metrics (except for number of states) were also well stratified between successful and nonsuccessful variants, particularly when considering measures based on estimated geographical distance; successful variants often doubled their geographic extent, with distances increasing by up to 1,000 km or more, whereas nonsuccessful variants frequently displayed no increase whatsoever (Figure 3(b) and Figures S14–S17). Such a high increase in geographic extent likely implies that successful variants are ones that have jumped between major swine-producing regions [13].

A Venn diagram visualized by the R's Vennerable v.3.0 package [61] was used to tabulate the number of variants that achieved success in one or more of the population, geographic, or genetic diversification aspects (Figure 3(c)). Interestingly, across scenarios, more than half (53%–67%) of the successful variants in the population aspect (based on any of the population measures) were also successful based on their geographic dispersion. In contrast, 48%–60% of the successful variants based on genetic diversification did not successfully expand geographically or population-wise. Only 3%–5% of successful variants achieved “success” across all three aspects. With that being said, most variants were not successful in any aspect (68%–74%) or in only one aspect (17%–23%) (Figure 3(c) and Figure S18).

Across the different combinations of candidate early indicators, measures of success, and temporal scenarios, 96 conditional logistic regression models were fitted on the training datasets. Only 41 models were considered significantly better than a null model (*p* < 0.05) based on the likelihood ratio test. From these models, we focused subsequent predictive modeling on the success measures that yielded the highest mean concordance (model fitting on the training set) and balanced accuracy (prediction on the test set, i.e., 2019–2020 data) for population expansion, spatial distribution, and genetic diversification. For population expansion, spatial distribution, and genetic diversification, the selected success measures were absolute increase in number of taxa, relative increase in maximum between-county geodesic distance, and absolute increase in 95^th^ percentile pairwise nucleotide distance, respectively. According to the concordance and balanced accuracy metrics, utilizing data from the previous 12 months provided the overall most accurate predictions for all the selected successes in the subsequent 12 months. Similarly, using the data from the previous 24 months generally yielded the highest predictive performance for most successes in the following 24 months, except for the model predicting the maximum between-county geodesic distance which had better performance when trained and tested on the previous 12 months of data to predict outcomes in the subsequent 24 months (Table 1). Thus, of all models fitted as part of this analysis, six models are presented in Table 1, focusing on the three selected success metrics and primarily the 12-*t*-12 short-term and 24-*t*-24 long-term scenarios. Across these models, concordance for the training data was >0.7 in all cases and balanced accuracy for predictions on new data ranged from 0.58 to 0.79. Predictive performance of other models was not as high (outputs of all models shown in Table S1).

The multivariate conditional logistic regression analysis showed that only one or two predictors out of the five examined were significantly associated (*p* < 0.05) with success in each model. Raw odds ratios are based on a 1 unit change in the predictor variable, whereas the entire range of many of our variables was far less than one. To make odds ratios more interpretable based on the observed range of each predictor, we calculated an adjusted odds ratio based on upper and lower quartiles of each predictor in the training data, while keeping other predictors constant. This can be interpreted as how many more times a variant is to be successful when moving from the first to the third quartile values of an early indicator variable. For population expansion, variants in the upper quartile for ancestral LBI had approximately 12–13 times higher odds of being successful in the next 12 or 24 months compared to variants in the lower quartile.

Regarding spatial distribution, variants with slower ancestral substitution rate (first quartile: 1 × 10^−3^ substitutions/nucleotide site/year (s/n/y)) had a greater chance of successfully extending their geographic distribution (maximum between-county geodesic distance) in the next 12 months compared to variants with higher estimated substitution rates (third quartile: 7 × 10^−3^ s/n/y). When focusing on a 24-month rather than 12-month follow-up period, there was a significant association between a variant's branch length and amino acid distance and the odds of increasing its geographic extent. Moving from the lower quartile (ancestral branch length of 0.003 or approximately 1.8 nucleotides diverged from its next phylogenetic common ancestor) to the upper quartile (0.017 or approximately 10.3 nucleotides diverged) quartiles of observed ancestral branch length, the odds of being the successful variant decreased by a factor of 10. Conversely, transitioning from the lower (0.025 or approximately 5 amino acids different from the most prevalent PRRSV-2 GP5 sequence) to the upper (0.075 or approximately 15 amino acids different) quartiles of observed ancestral amino acid distance resulted in 14.5 times greater odds of being a successful variant.

Genetic diversification (absolute increase in 95^th^ percentile pairwise nucleotide distance) showed a significant association with branch length in the short-term period and with amino acid distance in the long-term period. Specifically, variants with an ancestral branch length of 0.018 (upper quartile approximately 10.9 nucleotides diverged from its inferred phylogenetic ancestor) had 4.9 times higher odds of experiencing high levels of genetic diversification in the next 12 months compared to variants with a length of 0.003 (lower quartile approximately 1.8 nucleotides diverged). Moreover, variants with an ancestral amino acid distance of 0.075 (upper quartile approximately 15 amino acids different from the most prevalent PRRSV-2 GP5 sequences) were 6.8 times more likely to undergo diversify in the following 24 months than variants with a distance of 0.03 (lower quartile approximately six amino acids different) (Table 1 and Figures S12, S16, and S17).

The best fit models displayed fair to good predictive performance on the test set, depending on the success aspect and scenario. Notably, the prediction of genetic diversity variant success in the next 12 months achieved the highest balanced accuracy and F1 score (BA = 0.79, F1 = 0.62), followed closely by the prediction of success in population expansion over the next 24 months (BA = 0.75, F1 = 0.57). Moreover, the prediction of variant success in population expansion within the next 12 months, as well as success in spatial dispersion and genetic diversity within the next 24 months, exhibited similar performance (BA = 0.67, F1 = 0.5). Predictions of spatial success over a 12-month timeframe were notably poorer in comparison (BA = 0.58, F1 = 0.43) (Table 1). When comparing model performance on predictions made on all the matched variants observed throughout the study period to model performance on the test set, it was found that the balanced accuracy of all the models was slightly lower (BA = 0.56–0.74). However, the F1 score decreased by more than half (F1 = 0.15–0.27) due to a high proportion of false positives (Figure S19 and Table S2). For unmatched variants for which the future success could not be assessed, the models predicted that 21%–28% of these would be successful in terms of genetic diversity, while 59%–68% of the variants were predicted to be successful in either population or geographic expansion (Figure S20).

## 4. Discussion

In this study, we utilized over 10 years (2010–2021) of PRRSV-2 ORF5 sequencing data representing PRRS circulation across the U.S. to retrospectively evaluate the predictability of emerging variants across time. Each phylogenetic-based variant was systematically traced through time over both short-term (12 months) and long-term (24 months) periods in order to first calculate putative early indicators from retrospective phylogenetic inference and then quantify various aspects of epidemiologic success during the follow-up period. Primarily, we found that variants that were classified as successful through population growth were also likely to be successful through geographic expansion, but typically did not show notable genetic diversification. Across all models presented in Table 1, early indicators that were significantly associated with variant success at least once included LBI, branch length, mutation rate of the ancestral branch, and amino acid distance from the most prevalent contemporary GP5 sequences. The best predictive performance was achieved in the models that predicted long-term population growth using LBI and short-term genetic diversification using ancestral branch length. When applied to new data, these models successfully captured a significant number of successful variants with good sensitivity though relatively low specificity. PPVs of the models were poor, as many predicted successful variants turned out to be false positives.

In general, virus emergences can be assessed using diverse criteria, including abundance, adaptability, host range, and diversity [62]. For example, reproductive success, gauged by an effective reproduction number (R_e_) above 1, indicates a virus's ability to emerge and spread in a population [63, 64]. Thus, measures of emergence success commonly employ prevalence and growth rate for predictions [35–37]. For PRRSV-2, measuring the impact of the emergence of novel variants goes beyond case numbers and should encompass spatial spread and variant genetic diversity, which play a crucial role in determining disease control efficacy. Our study showed emerging variants, as indicated by higher detection rates (population increases), also displayed extensive geographic expansion but lacked substantial genetic diversification within the given timeframe. These findings align with the idea that highly abundant variants have a greater likelihood of geographic dissemination compared to others, especially through routes such as animal transport [65, 66].

Various parameters such as LBI [35], epitope features [36], phenotypic data [67], and consensus sequence of the current viral population [68] have been shown to be important in forecasting seasonal influenza A virus (IAV). However, it remains unclear whether these early indicators are useful when applied to PRRSV-2 data, as these two viral species differ significantly in their evolutionary dynamics. For instance, IAV branching patterns, which affect LBI values, are shaped by short-lived viral variants that quickly go extinct, and selective sweeps caused by frequent antigenic drift in IAV, resulting in a comb-like or ladder-like genealogy tree [69, 70]. In contrast, the persistent cocirculation and sequential dominance of various PRRSV-2 subpopulations [7, 13] give rise to phylogenetic clades with a bush- or star-like structure (short internal branches and long external branches). Nevertheless, our analysis demonstrates that LBI was the only indicator with significant predictive power in forecasting both short-term and long-term population expansion of PRRSV-2 in the best fit models. This suggests that the underlying assumption of LBI, which is that rapid branching patterns are associated with high fitness of the inferred ancestor [35], may also apply to PRRSV-2 phylodynamics.

Understanding of immune epitopes, antigenic properties, and genotype–phenotype relationships of PRRSV-2 remains incomplete and cannot be directly inferred from sequencing data [71–73]. This lack of knowledge makes it challenging to incorporate such features into prediction models. To address this issue, we developed two indicators to capture the putative distinctiveness of a variant compared to the current most prevalent GP5 protein at a given point in time: GP5 amino acid distance and N-glycosylation pattern similarity. The GP5 amino acid distance parameter is relatively similar to the best predictor used to forecast IAV reported by Barrat-Charlaix et al. [68]. Variants with more divergent GP5 proteins were >6 times more likely to become more genetically diverse, and >14 times more likely to undergo spatial expansion than less divergent variants. We hypothesize that this metric captures, in part, the extent to which epitopes found on GP5 may differ from those recognized by the prevailing immunity in the population, hence more divergent variants may be able to better evade preexisting immunity at the population level. That being said, the nature of our data did not allow us to know whether the emerging variants infected the same animals or farms that were previously exposed to the prevailing GP5 protein.

N-glycosylation pattern similarity parameter focuses on specific amino acid sites involved in potential glycan shielding, which is one immune evasion mechanism utilized by PRRSV-2 [74, 75]. These sites have evolved under positive selection pressure [12, 76–78] and are believed to be associated with emergence at the sublineage level [7, 79]. However, N-glycosylation pattern similarity was not significantly associated with a variant's success in any of our predictive models. Although previous work suggested that N-glycosylation pattern changes sometimes coincided with PRRS epidemic events, the patterns were not stable within a sublineage (only 40%–60% of sequences in sublineage shared a N-glycosylation pattern) [79]. Thus, N-glycosylation patterns may change too frequently to attribute a single pattern to a particular variant, as we did here.

Branch length of the ancestral node was the sole significant predictor in the best fit model predicting success in genetic diversity in the short-term. Specifically, variants whose inferred ancestors had undergone greater evolutionary changes (longer branch lengths) were more likely to genetically diversify shortly after. We hypothesize that these rapidly evolving variants had not yet reached a state of fitness stability and hence continued diversifying during the early stage of emergence. Ultimately, the disparity in significant predictors between the short-term and long-term models, along with the notably superior performance of the short-term model, led us to conclude that branch length stands out as the most robust predictor for success in genetic diversity. Branch length was also a significant predictor alongside GP5 amino acid distance in the model predicting spatial success in long-term. The association of branch length to the spatial success (OR < 1) was opposite to its association to the genetic success (OR > 1) which was consistent with the observation that variants that expand geographically did not tend to undergo substantial diversification (Figure 3).

An essential task for this research was defining a PRRSV-2 variant. Given the large dataset and the need to build numerous trees with different subsets of data, the primary phylogenetic tree-building method we utilized (FastTree ML) was the most plausible approach, offering an adequately reliable overview of the genetic relationships among the viruses, albeit not the most accurate tree-building approach [44, 80]. Accordingly, variant classifications based on the tree's patristic distance (TreeCluster's Avg Clade [46]) may differ if an alternative tree-building method had been used. In our analysis, we used an average patristic distance cut-off of 2% to define variants, which proved suitable because the variant size and clade support remained consistent across various trees and scenarios and are in-line with thresholds conventionally used to define PRRSV sequences as homologous or heterologous [81]. However, this approach has limitations as it can lead to abrupt appearance of new variants in the follow-up period that appear to be >2% from any of the original sequences. New variants accounting for approximately 5%–25% of total variants per time period. These occurrences could potentially signify the rapid emergence of new variants (<6 months), the introduction of exotic variants, or the reemergence of under-the-radar variants absent from the current sequencing data.

The estimation and prediction of PRRSV-2 variant emergence was based on phylogenies constructed with the ORF5 gene sequence, which constitutes only about 4% (603 nucleotides) of the entire genome spanning approximately 15,000 nucleotides. Several studies have underscored that genome-based epidemiological investigations offer a clearer understanding of PRRSV-2 evolution, particularly concerning emergence events facilitated by genomic recombination [19, 82–84]. However, whole-genome sequencing of PRRSV-2 is sporadic and usually reserved for atypical PRRSV-2 cases or experimental studies. In contrast, the generation of the ORF5 sequence occurs routinely. Additionally, phylogenetic tree topologies derived from both the ORF5 sequence and the whole genome exhibit relative similarity [18], particularly for clades that have a very recent common ancestor (i.e., <2% genetic distance, which was the threshold used here). Consequently, ORF5 sequences currently represent the best opportunity for predictive modeling; if the availability of whole genome sequences in surveillance datasets improves, incorporation of whole genome data could enhance the performance of our models.

Another limitation of this research relates to the interpretation of geographic expansion. Numerous external factors (i.e., animal movement) contribute to the spatial dissemination of a virus that is not measurable from viral phylogenies nor related to a virus's phenotype. In addition, sampling locations were not known for all sequences, and not all pig-producing regions of the U.S. were equally well represented in the dataset (Figure S1). On the other hand, spatial and/or temporal stratified downsampling of sequences prior to predictive modeling may have resulted in missing some actual rapid population or geographical expansions. More comprehensive spatial data could improve model predictions, as could incorporating regional variability in the prevailing ORF5 sequences and emergence success.

We identified certain early indicators that are associated with predicting various aspects of success. However, when implementing these models to all matched variants beyond the selected variants in the case-control design, one key issue undermining model performance was the low PPV. This was unsurprising, given that the training case-control dataset had a significantly higher proportion of successes than the overall data. Because of the low PPV, predictions of variant success could be better interpreted as identifying those variants with high emergence potential (with not all variants realizing their potential) as opposed to a projection of what will happen with certainty. In addition, population and spatial emergence success was commonly predicted for mismatched variants, whose actual success could not be measured. If predictions are generated prospectively, we suggested filtering out such variants by removing variants with low clade support (<75%) before making predictions. This strategy could enhance both PPV and overall accuracy.

Despite the low PPV, our models successfully identified a variant with nine taxa in the initial tree (based on 12 months of data from 2019–2020), which ultimately led to the impactful emergence of the novel L1C-1-4-4 outbreak in the Midwestern U.S. This prediction was made as early as January 2020, more than 6 months prior to the first official notice of the outbreak in fall 2020 [10]. The models correctly anticipated that this variant would exhibit both population growth and geographic expansion but would not undergo significant genetic diversification, aligning with our hypothesis of a high-fitness variant.

Nevertheless, we acknowledge that our work represents just the initial stage of developing methods for prediction of the emergence of PRRSV-2 variants, highlighting informative phylogenetic-based early indicators for a variant's emergence. While this predictive modeling benefited from long-term nationwide data, the best fit models and the highlighted key early indicators discussed herein may not necessarily generalize to future instances of PRRSV-2 emergence. This limitation arises due to the rapid dynamics observed in the PRRSV-2 population and its spatial distribution [7, 13]. Hence, it is crucial to continue improving our approach by incorporating better spatial-related metadata, expanding the training and the test sets with more data in the future, and exploring additional potential predictors. Our ability to predict PRRSV-2 variant emergence holds significant promise in advancing future PRRS control and prevention strategies. This predictive capacity serves as a valuable tool for early detection of variants of interest or concern, enabling targeted interventions like prompt biocontainment at affected premises or the development of updated vaccines tailored to potential emerging variants in subsequent seasons.

## 5. Conclusions

This is the first study to systematically analyze and evaluate predictive modeling for PRRSV-2 variant emergence. Our findings revealed that variants which had successful population growth also tended to expand geographically, often without significant genetic diversification. LBI was consistently found as the only early indicator in models predicting both short-term and long-term population expansion. Meanwhile, ancestral branch length was strongly associated with short-term genetic diversification, and the GP5 amino acid distance was linked to long-term success in both geographic distribution and genetic diversity. Low PPVs were found when the predictive models were applied to variants not included in the case-control design. However, false positives are not necessarily meaningless in this context. Indeed, such false positives may represent variants that share many features of successful variants and could be considered as variants at-risk of emergence. Thus, model predictions could be interpreted as providing insights into which variants have high emergence potential, as opposed to strictly those that will emerge. However, further improvements are necessary, accompanied by filling knowledge gaps in PRRSV-2 immuno-epidemiology, to determine how best to implement these predictions for enhanced prevention and control of PRRS.

## Figures and Tables

**Table 1:** The best fit model for each success aspect and predicted period.

Success aspect	Measure of success	Scenario (model)	Model *p*-value (LRT)	Predictor	*p*-value	Coefficient	Raw OR	Adjusted OR	Predictor's lower upper quartiles	Training set	Test set					
										Concordance	Sensitivity	Specificity	PPV^†^	NPV^††^	F1 score	Balanced accuracy
Population expansion	Absolute increase in number of taxa	12-*t*-12	0.002	Branch length	0.098	−1,423.0	7.9 × 10^−63^	0.12	0.00–0.02	0.875	0.75	0.58	0.38	0.88	0.500	0.667
				Average clade rate	0.808	−200.0	1.3 × 10^−87^	0.4	0.00–0.01							
				LBI	0.015^*∗*^	8.1	3.3 × 10^3^	12.12	0.03–0.333							
				Amino acid distance	0.159	20.9	1.1 × 10^9^	3.15	0.020–0.075							
				N-gly similarity	0.410	1.4	4.0	1.79	0.333–0.750							
		24-*t*-24	0.001	Branch length	0.120	−220.4	1.8 × 10^−96^	0.08	0.002–0.013	0.810	1.00	0.50	0.40	1.00	0.571	0.750
				Ancestral rate	0.446	268.9	6.1 × 10^116^	3.65	0.002–0.007							
				LBI	0.030^*∗*^	8.9	7.2 × 10^3^	12.67	0.000–0.286							
				Amino acid distance	0.652	7.7	2.2 × 10^3^	1.42	0.030–0.075							
				N-gly similarity	0.939	0.2	1.2	1.09	0.333–0.750							

Spatial distribution	Relative increase in maximum between-county geodetic distance	12-*t*-12	0.006	Branch length	0.587	19.9	4.3 × 10^8^	1.34	0.003–0.018	0.729	0.75	0.42	0.30	0.83	0.429	0.583
				Ancestral rate	0.035^*∗*^	−898.7	0	0	0.001–0.007							
				LBI	0.116	5.8	3.5 × 10^2^	6.04	0.026–0.333							
				Amino acid distance	0.725	5.9	3.5 × 10^2^	1.38	0.020–0.075							
				N-gly similarity	0.081	2.8	1.7 × 10^1^	3.24	0.333–0.750							
		12-*t*-24	0.029	Branch length	0.044^*∗*^	−170.7	7.5 × 10^−75^	0.1	0.003–0.017	0.792	0.50	0.83	0.50	0.83	0.500	0.667
				Ancestral rate	0.136	−309.4	4.3 × 10^−135^	0.17	0.002–0.008							
				LBI	0.293	5.5	2.5 × 10^2^	5.13	0.026–0.322							
				Amino acid distance	0.022^*∗*^	53.4	1.6 × 10^23^	14.47	0.025–0.075							
				N-gly similarity	0.620	0.8	2.2	1.39	0.333–0.750							

Genetic diversity	Absolute increase in 95^th^ percentile pairwise nucleotide distance	12-*t*-12	<0.001	Branch length	0.004^*∗*^	108.1	8.6 × 10^46^	4.92	0.003–0.018	0.917	1.00	0.58	0.44	1.00	0.615	0.792
				Ancestral rate	0.603	−141.6	3.2 × 10^−62^	0.42	0.001–0.007							
				LBI	0.758	−1.2	2.9 × 10^−1^	0.69	0.026–0.333							
				Amino acid distance	0.909	2.1	7.9	1.12	0.020–0.075							
				N-gly similarity	0.779	0.6	1.7	1.27	0.333–0.750							
		24-*t*-24	0.006	Branch length	0.119	62.1	9.4 × 10^26^	2.07	0.002–0.013	0.857	1.00	0.33	0.33	1.00	0.500	0.667
				Ancestral rate	0.880	−46.6	5.9 × 10^−21^	0.8	0.002–0.007							
				LBI	0.958	−0.2	8.2 × 10^−1^	0.95	0.000–0.286							
				Amino acid distance	0.045^*∗*^	42.5	2.9 × 10^18^	6.78	0.030–0.075							
				N-gly similarity	0.535	1.8	5.8	2.09	0.333–0.750							

^*∗*^*p* < 0.05, ^†^positive predictive value, ^††^negative predictive value.

## Data Availability

The additional nonsensitive data and analytic results used to support the findings of this study are all included within the supplementary information file.

## Supplementary Materials

Supplementary Materials Additional information and results from the analysis.
