# Supplementary material for: Predicting Potential PRRSV-2 Variant Emergence through Phylogenetic Inference
Source: Transbound Emerg Dis. 2024 Feb 5;2024:7945955. doi: 10.1155/2024/7945955 (PMC12017126; doi:10.1155/2024/7945955)
Supplement: Supplementary Materials — Additional information and results from the analysis. [file 7945955.f1.docx]

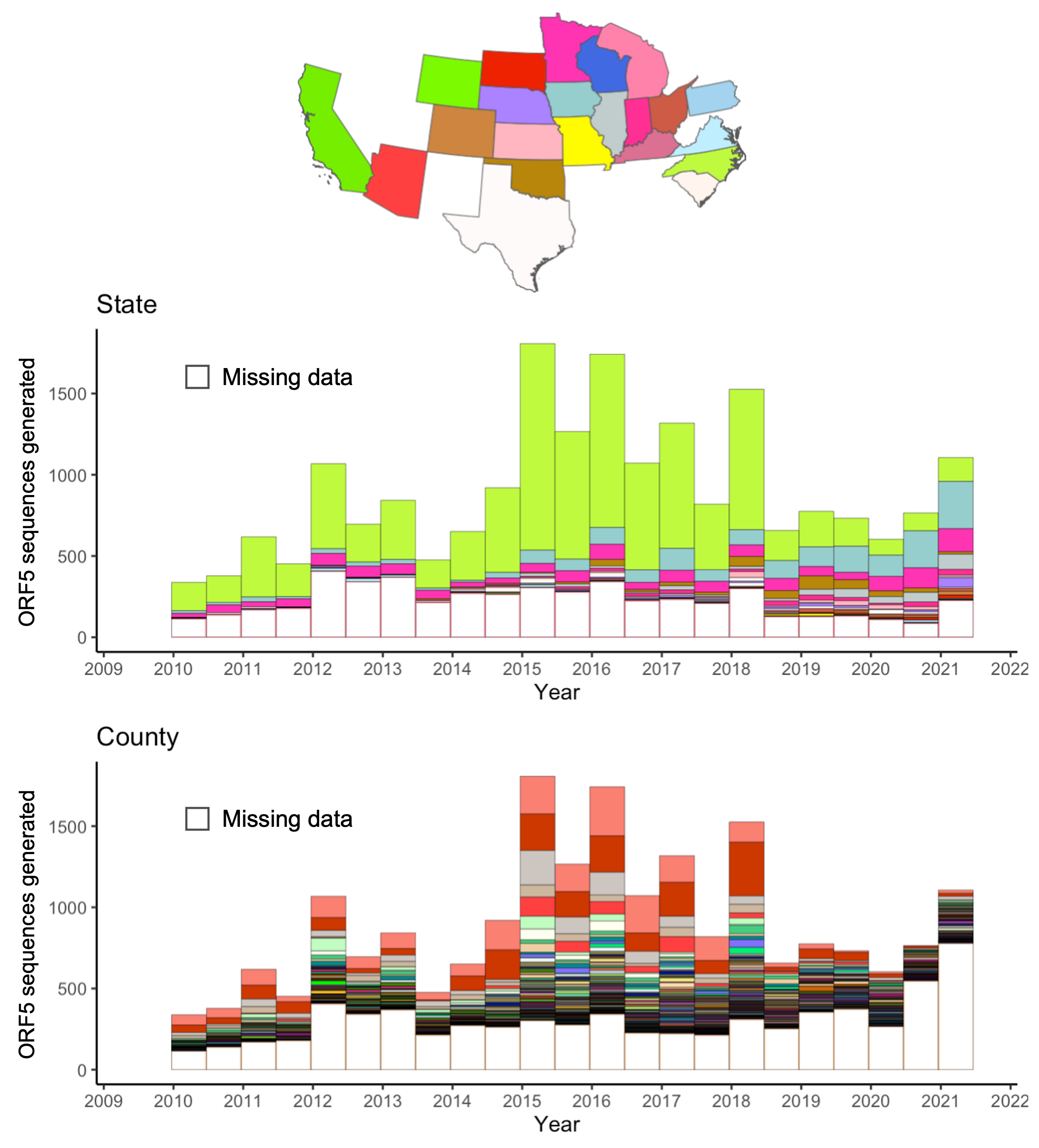


**Supplementary Figure S1.** Temporal distribution of PRRSV-2 L1 ORF5 sequencing data with spatial data (state and county) availability. Color of states in the top map correspond to colors in the distribution plot at state level (middle).


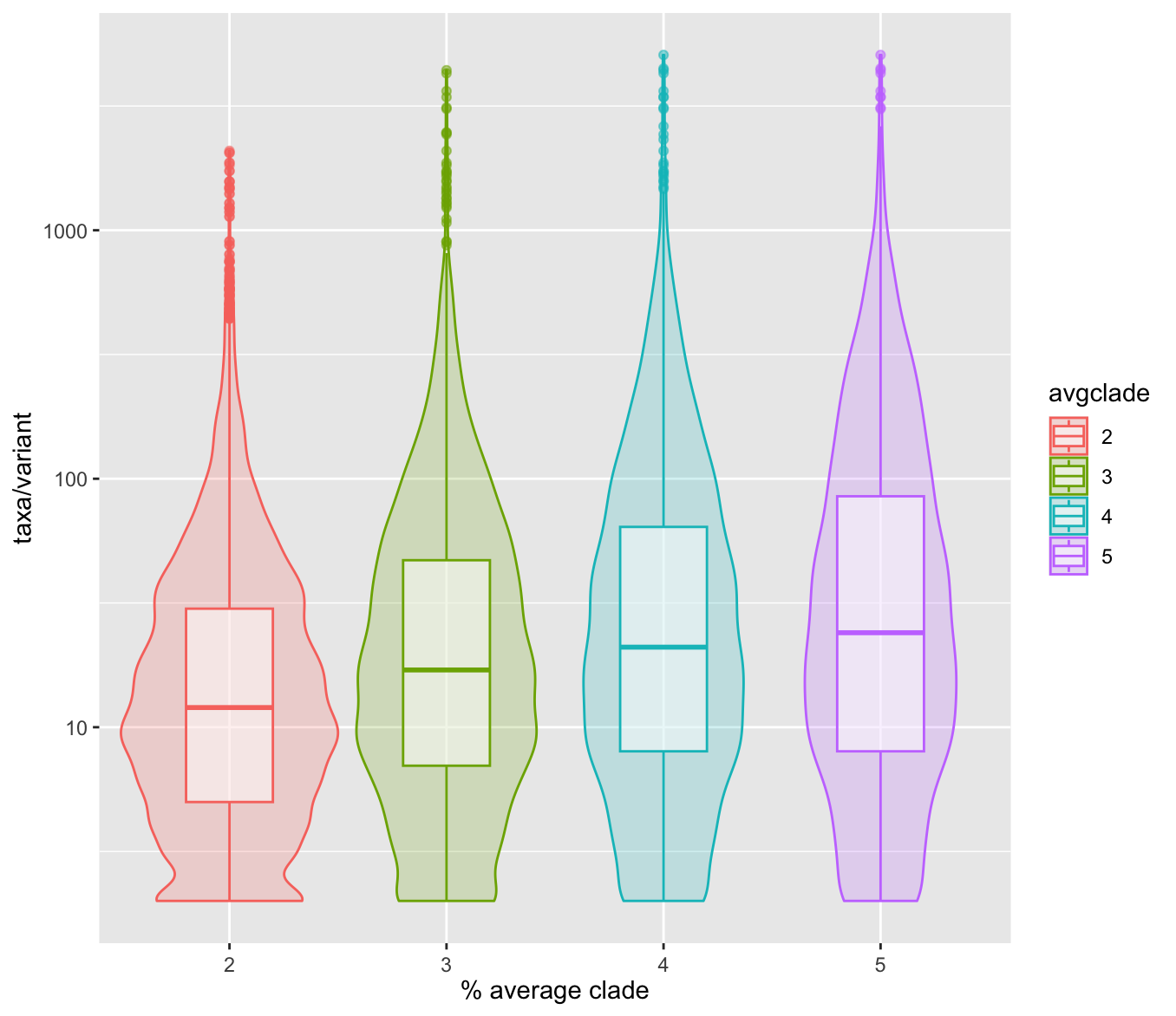


**Supplementary Figure S2.** Median number of taxa per variant defined by different % average patristic distance (TreeCluster’s Avg Clade).


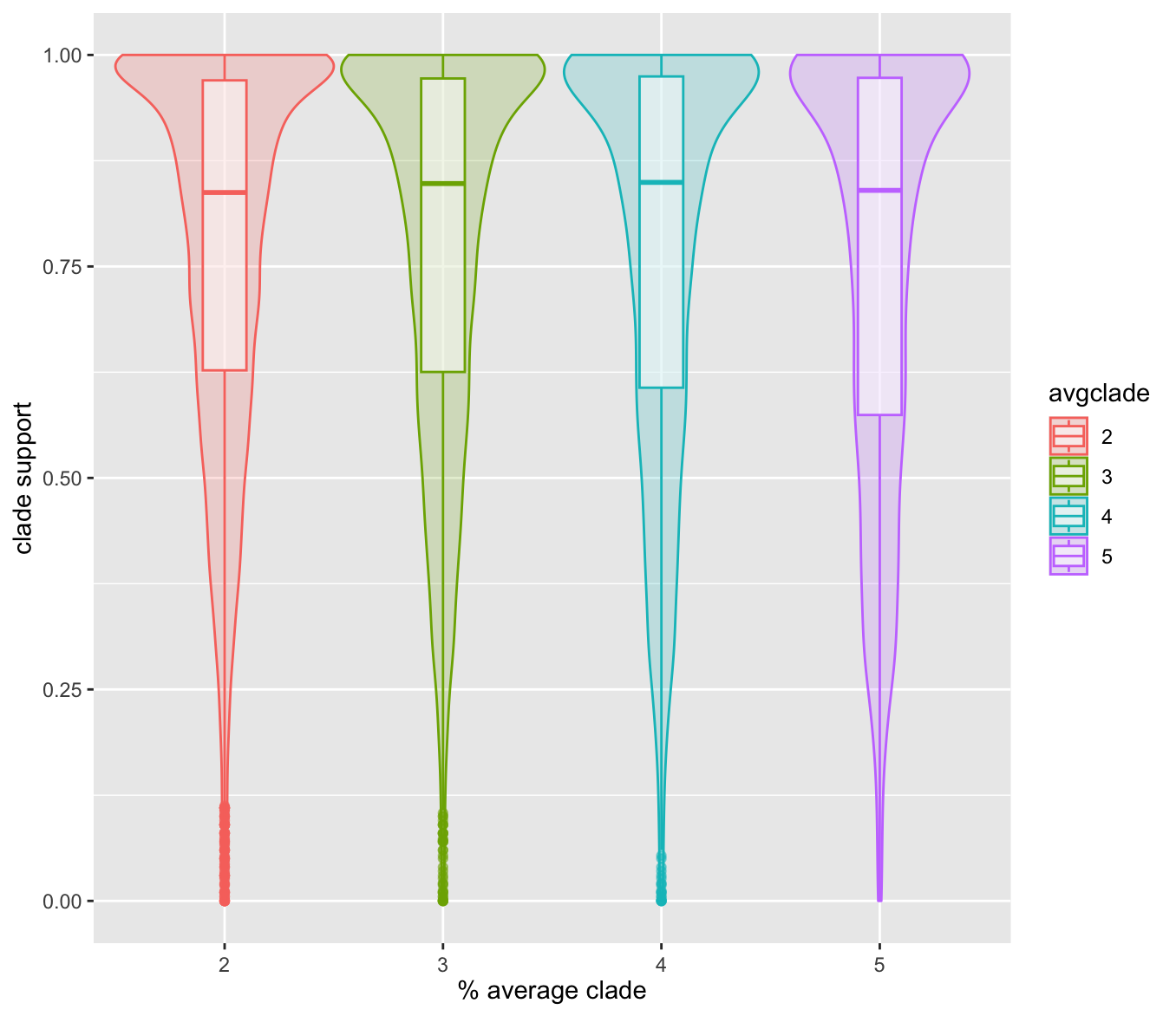


**Supplementary Figure S3.** Median bootstrap support of variant clade (variant defined by different % average patristic distance).


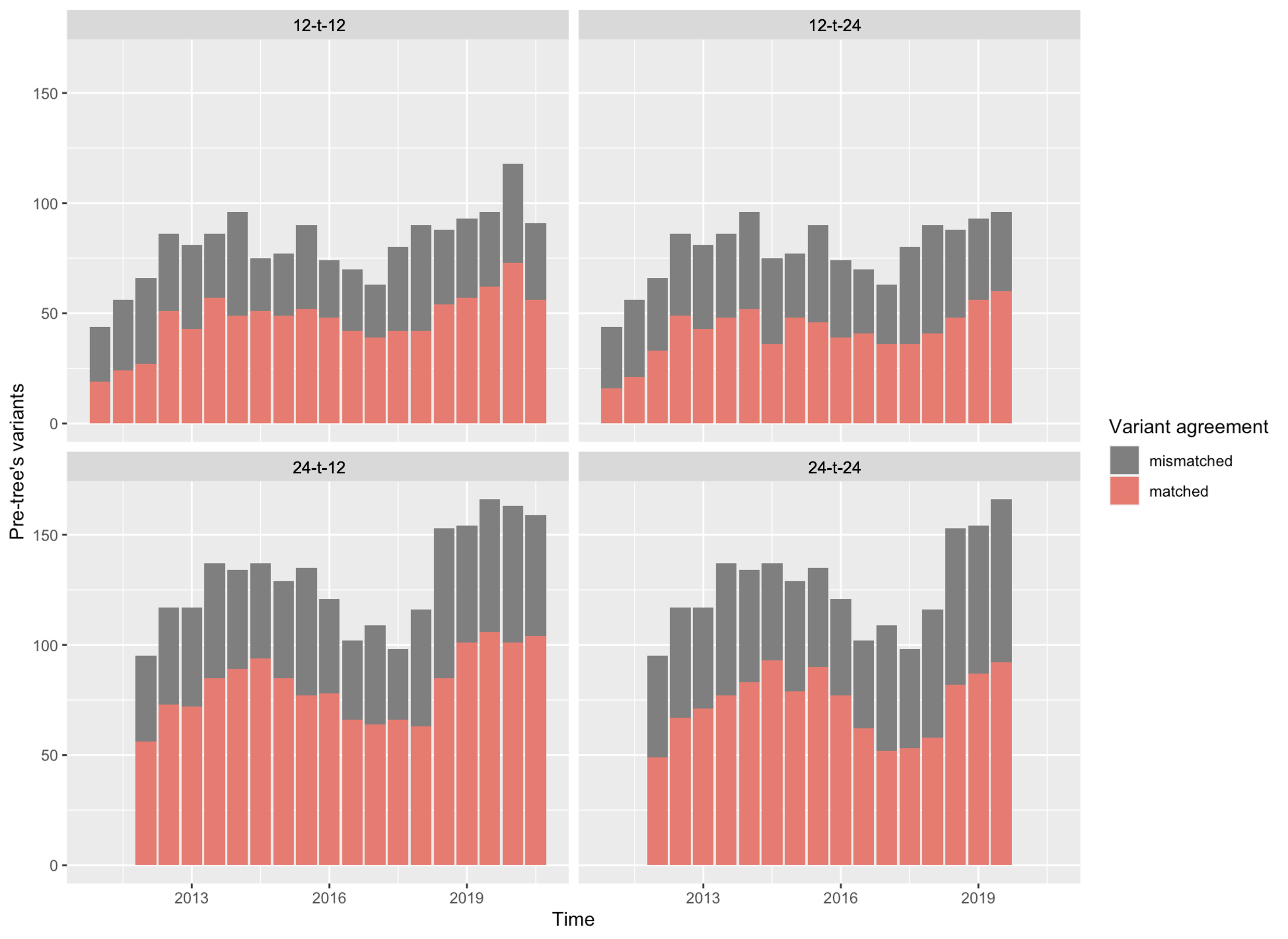


**Supplementary Figure S4.** Number of matched post-tree’s variants (red) across time of each scenario.


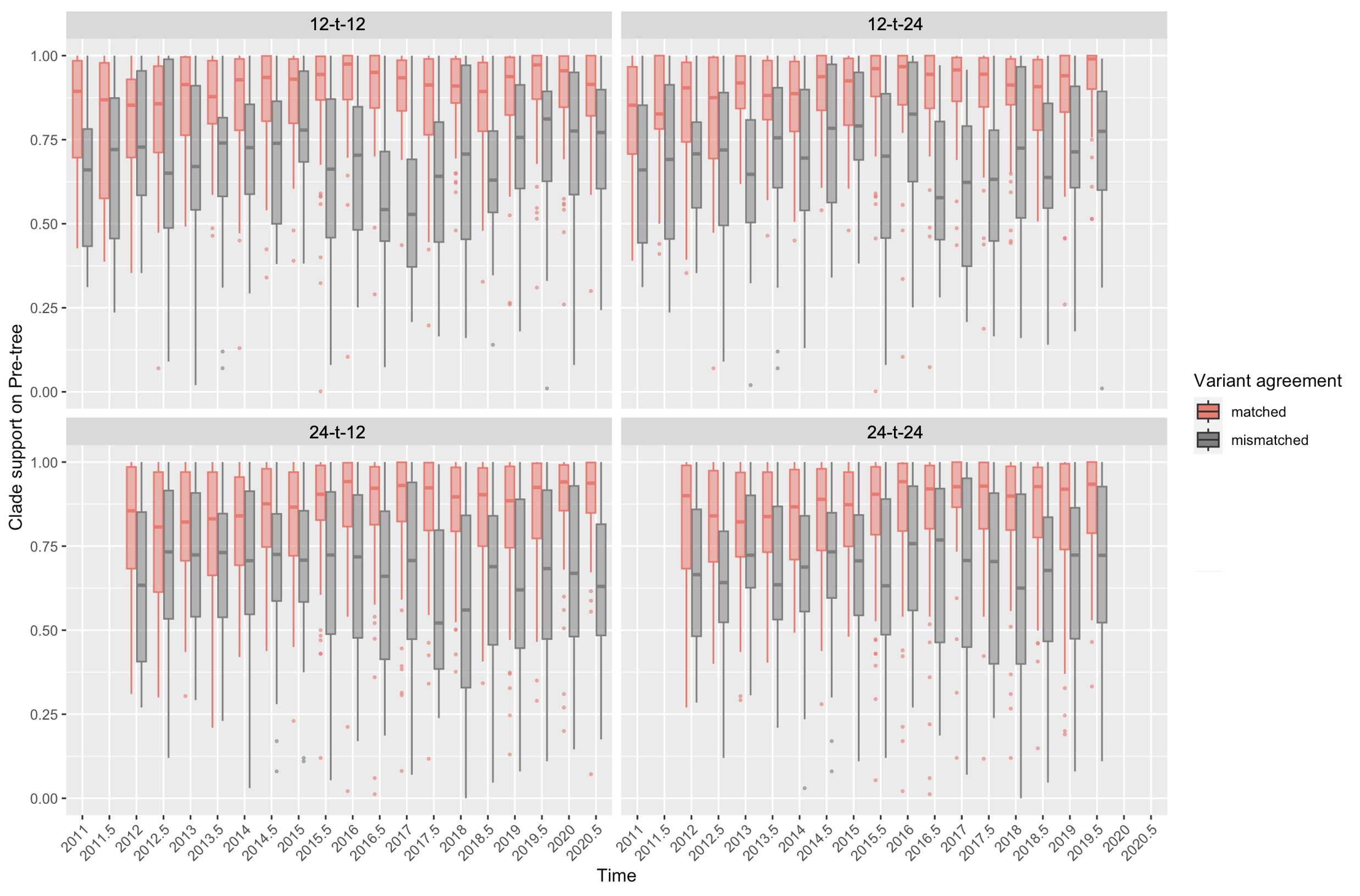


**Supplementary Figure S5.** Median bootstrap clade support of matched vs mismatched post-tree’s variants of each scenario.


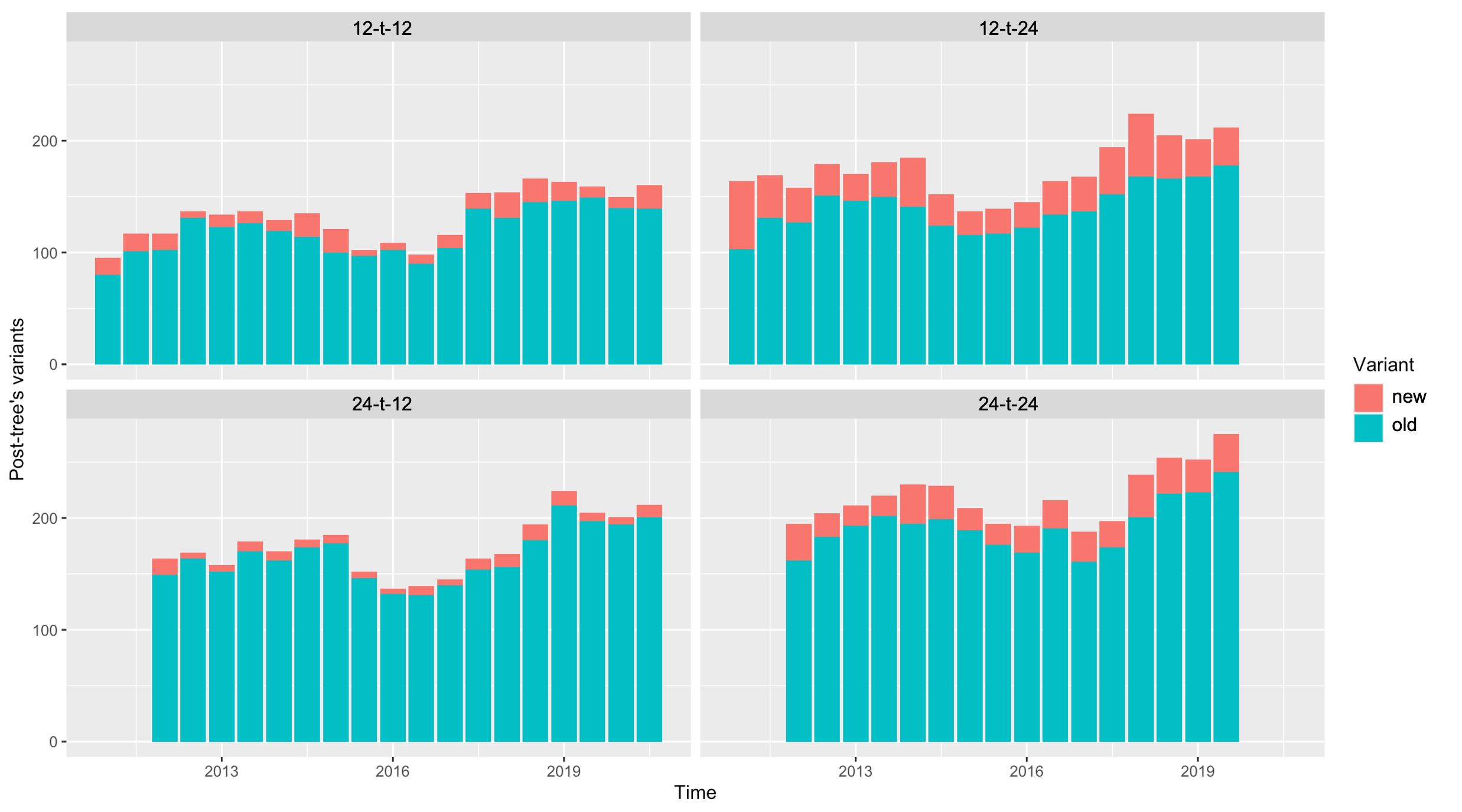


**Supplementary Figure S6.** Number of new variants found on post-trees (red) of each scenario.


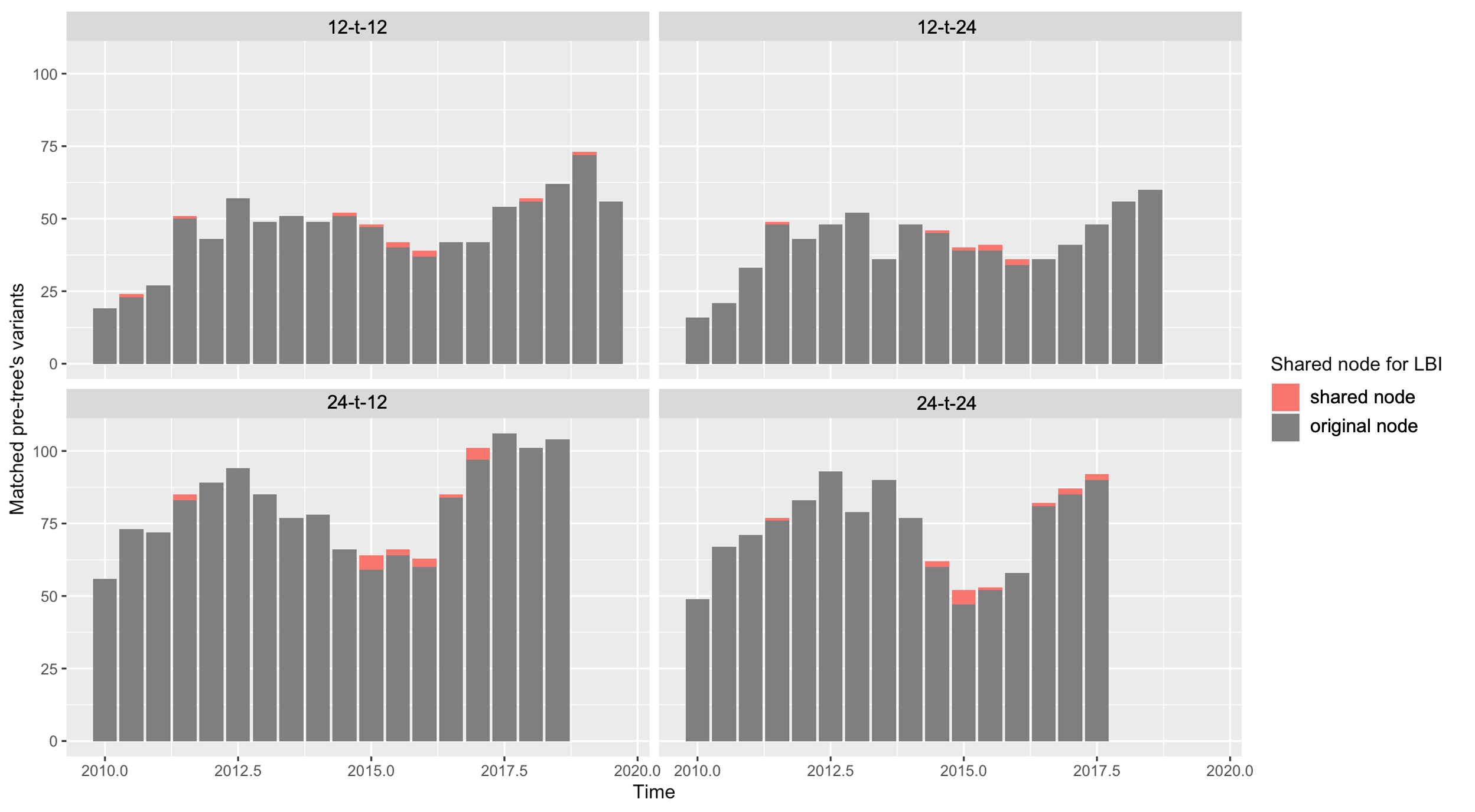


**Supplementary Figure S7.** Number of matched pre-tree’s variant sharing the same ancestral node (red) in pruned strict clock timed tree that used for local branching index (LBI) calculation.


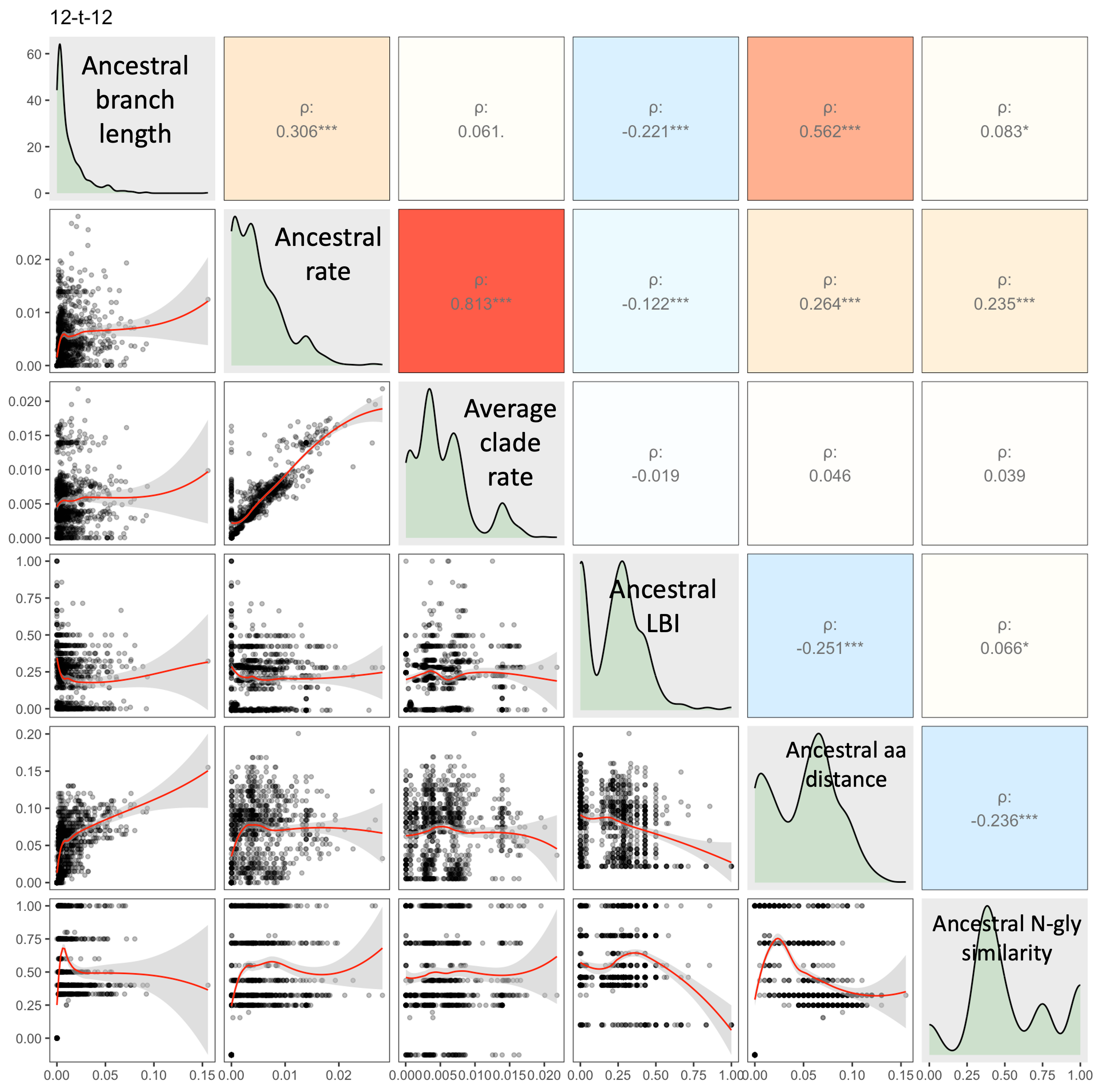


**Supplementary Figure S8.** Matrix of Spearman’s correlation coefficients (ρ) between all candidate early indicators with background color corresponding to the strength of correlation from 1 (red) to -1 (blue) (upper panel), their data density plots (diagonal), and scatterplots colored with LOESS curves fitted (red line) and associated 95% confidence intervals (grey polygon) (lower panel) for the 12-*t*-12 scenario.


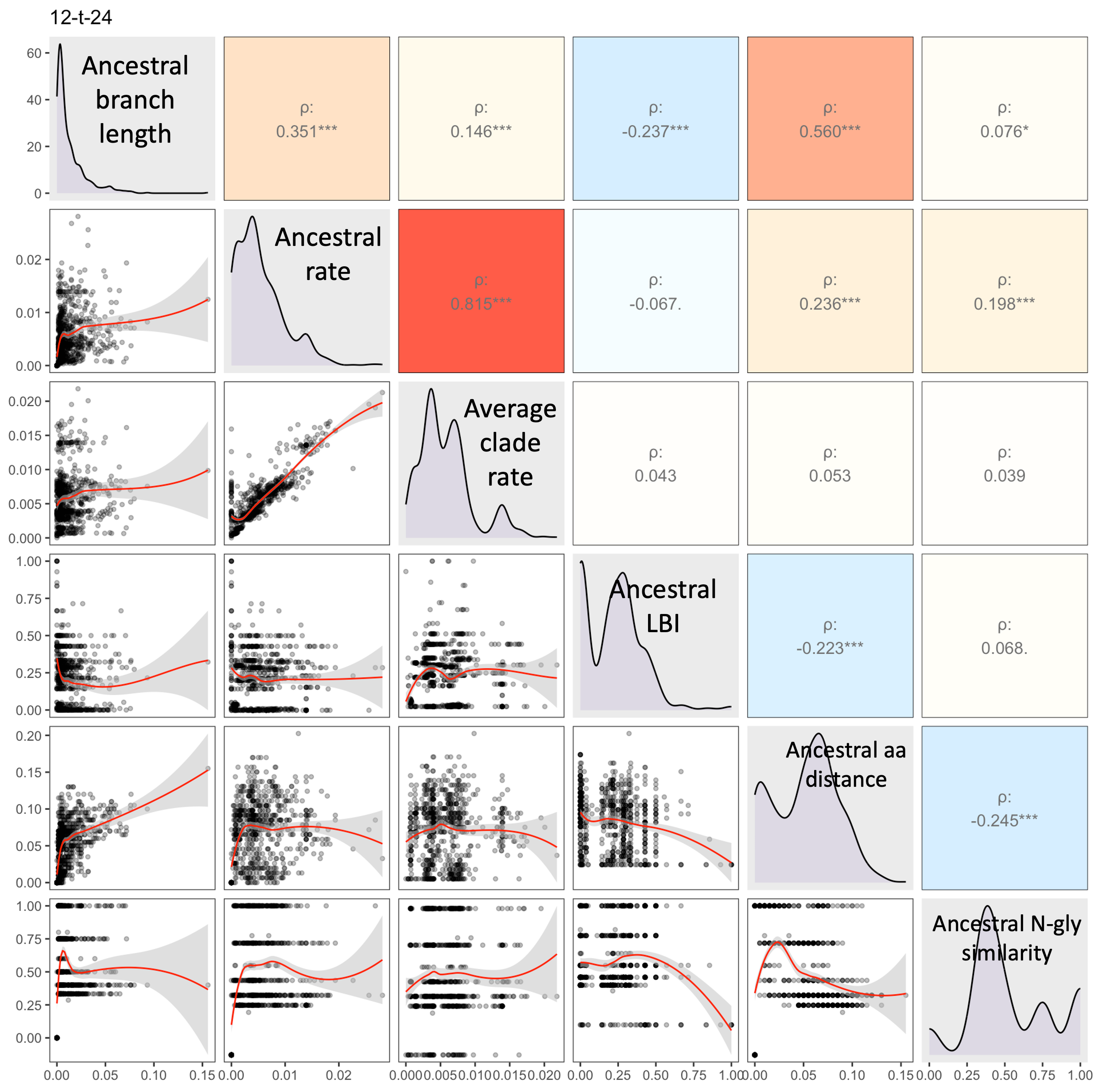


**Supplementary Figure S9.** Matrix of Spearman’s correlation coefficients (ρ) between all candidate early indicators with background color corresponding to the strength of correlation from 1 (red) to -1 (blue) (upper panel), their data density plots (diagonal), and scatterplots colored with LOESS curves fitted (red line) and associated 95% confidence intervals (grey polygon) (lower panel) for the 12-*t*-24 scenario.


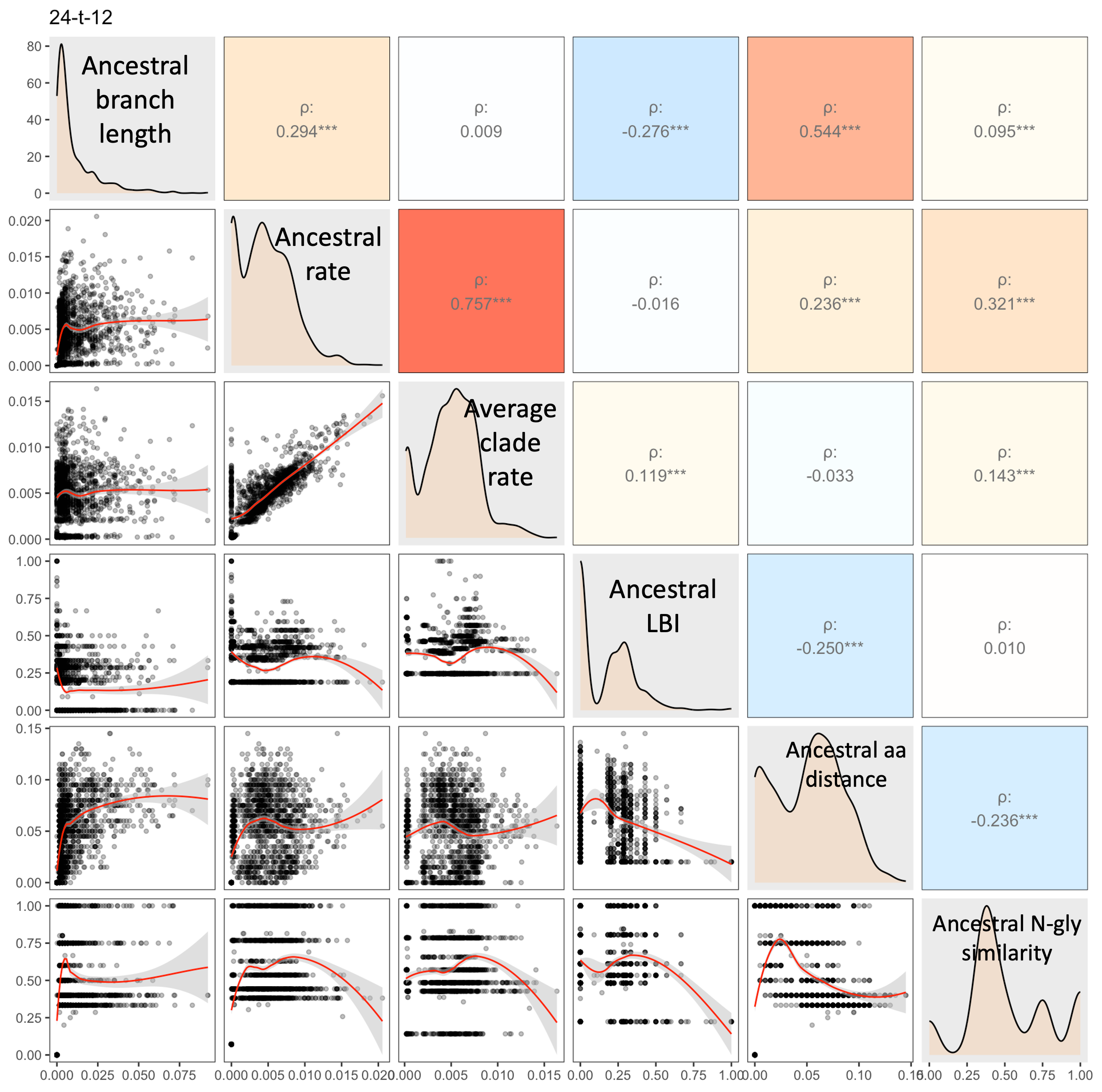


**Supplementary Figure S10.** Matrix of Spearman’s correlation coefficients (ρ) between all candidate early indicators with background color corresponding to the strength of correlation from 1 (red) to -1 (blue) (upper panel), their data density plots (diagonal), and scatterplots colored with LOESS curves fitted (red line) and associated 95% confidence intervals (grey polygon) (lower panel) for the 24-*t*-12 scenario.


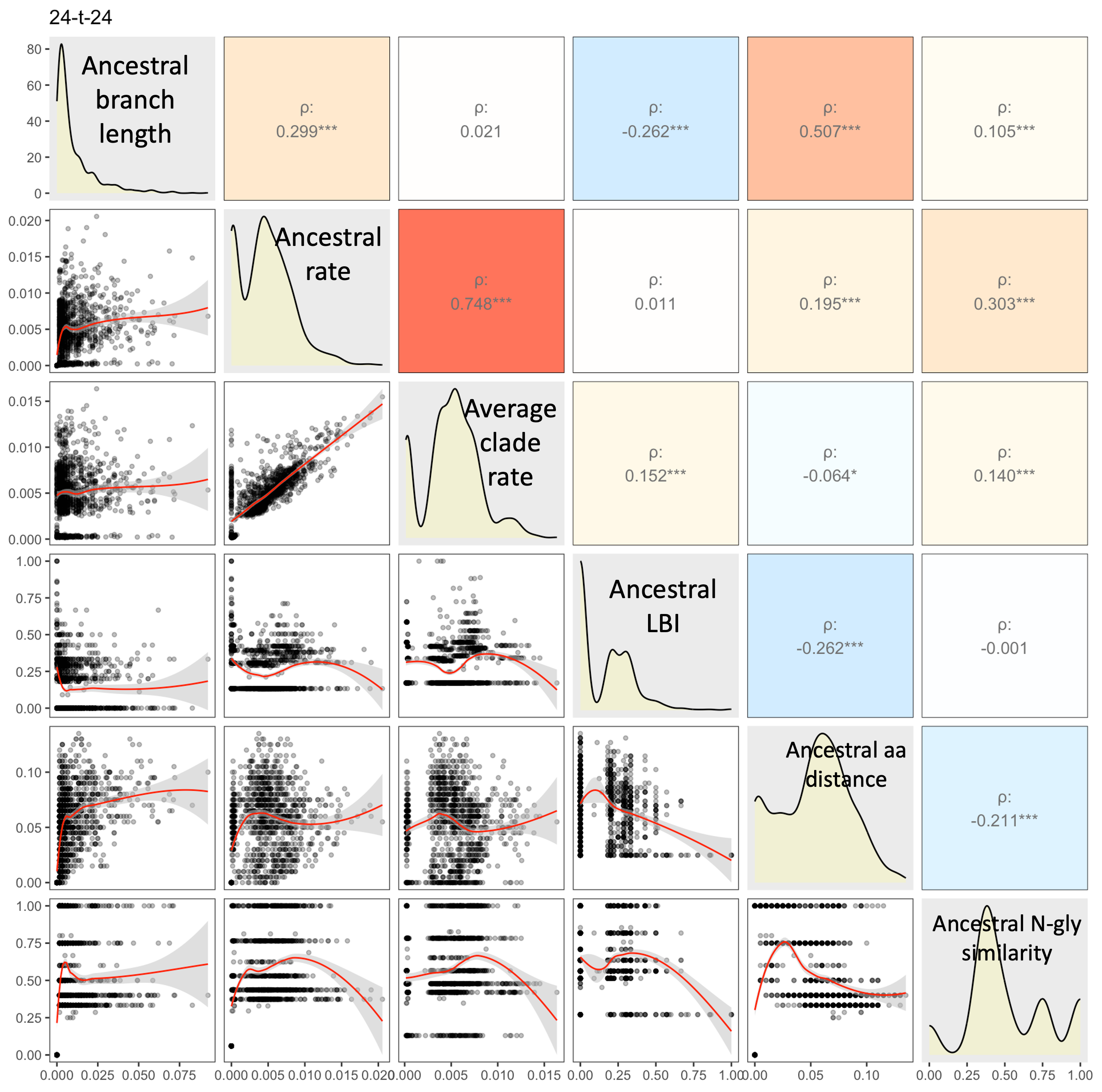


**Supplementary Figure S11.** Matrix of Spearman’s correlation coefficients (ρ) between all candidate early indicators with background color corresponding to the strength of correlation from 1 (red) to -1 (blue) (upper panel), their data density plots (diagonal), and scatterplots colored with LOESS curves fitted (red line) and associated 95% confidence intervals (grey polygon) (lower panel) for the 24-*t*-24 scenario.


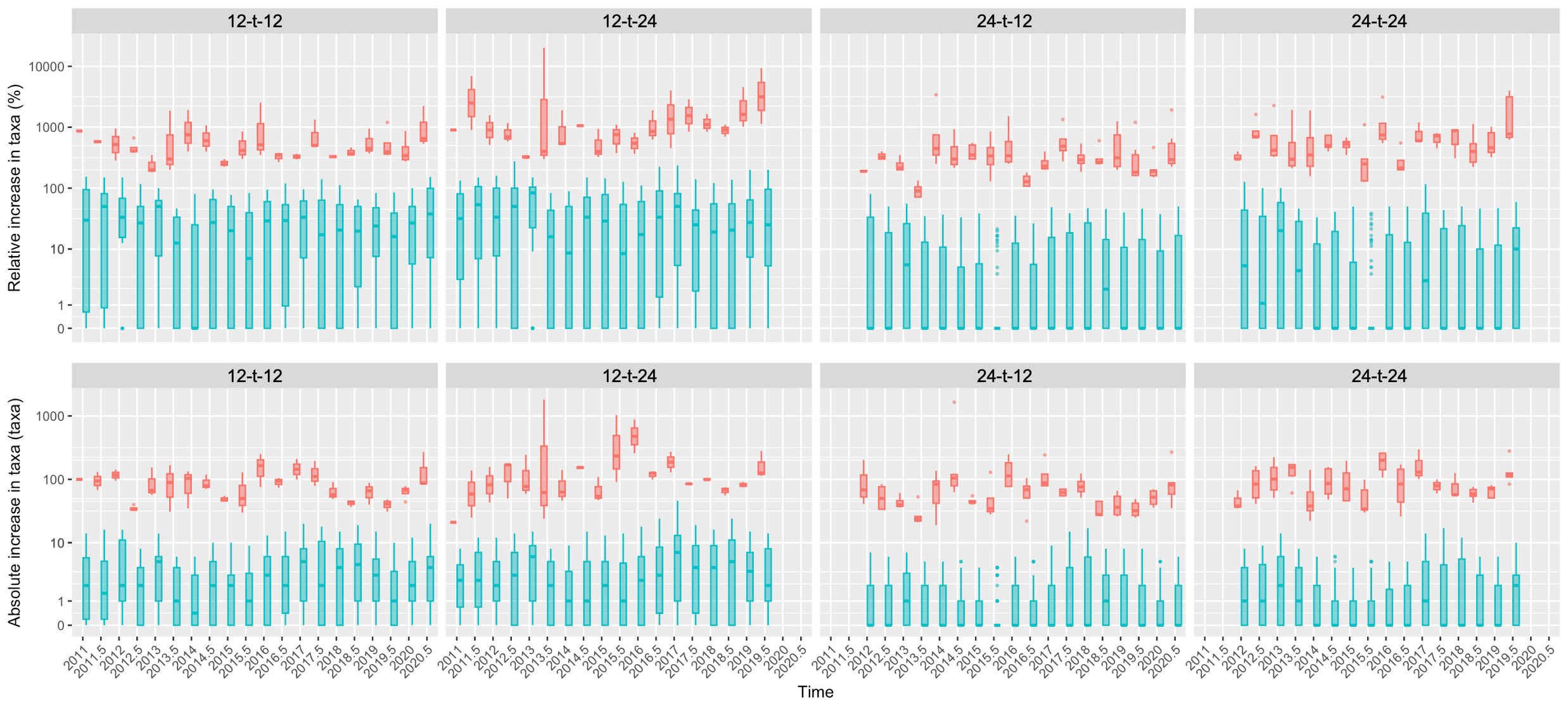


**Supplementary Figure S12.** Differences between success (>95^th^ percentile of each success measure: red) and unsuccess (<75^th^ percentile of each success measure: blue) of relative and absolute increases in number of taxa.


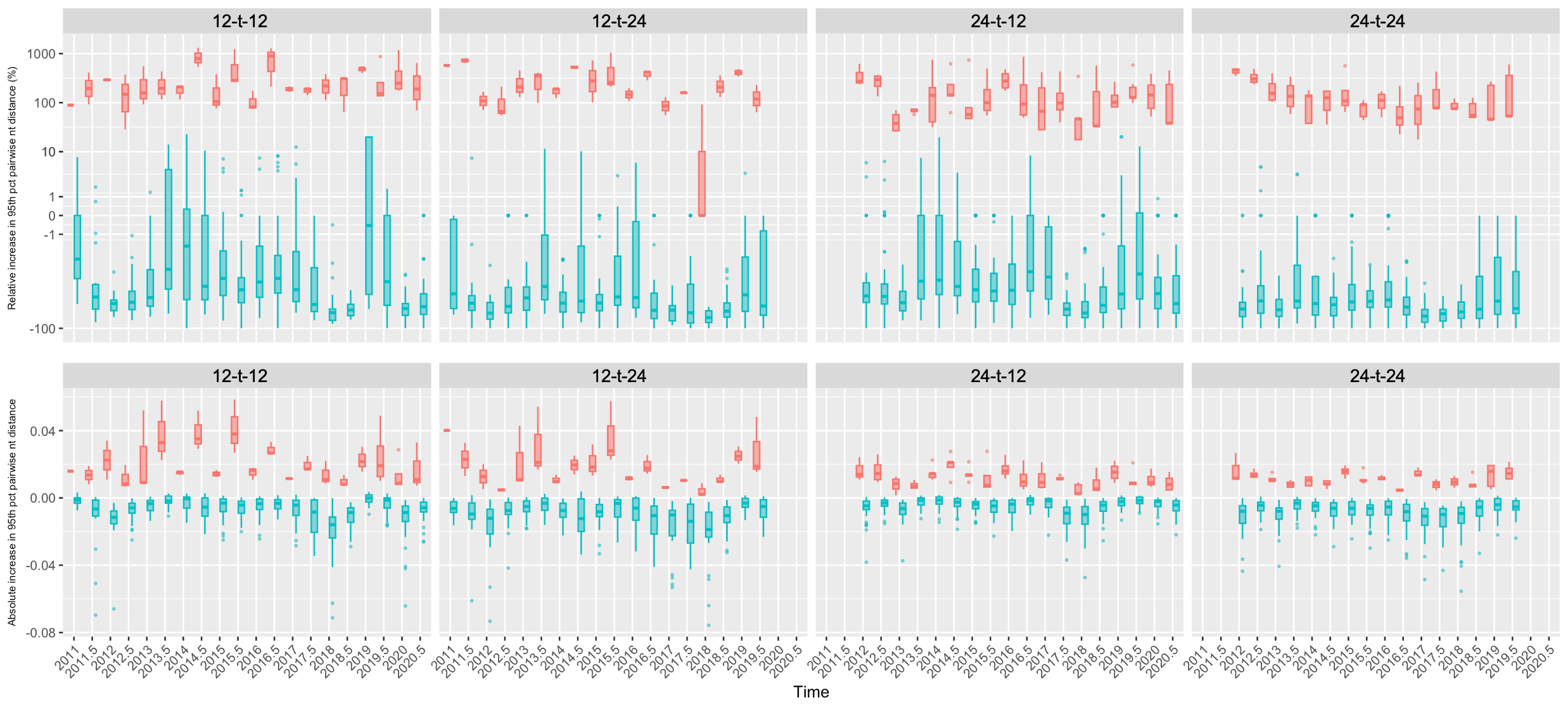


**Supplementary Figure S13** Differences between success (>95^th^ percentile of each success measure: red) and unsuccess (<75^th^ percentile of each success measure: blue) of relative and absolute increases in 95^th^ percentile pairwise nucleotide distance.


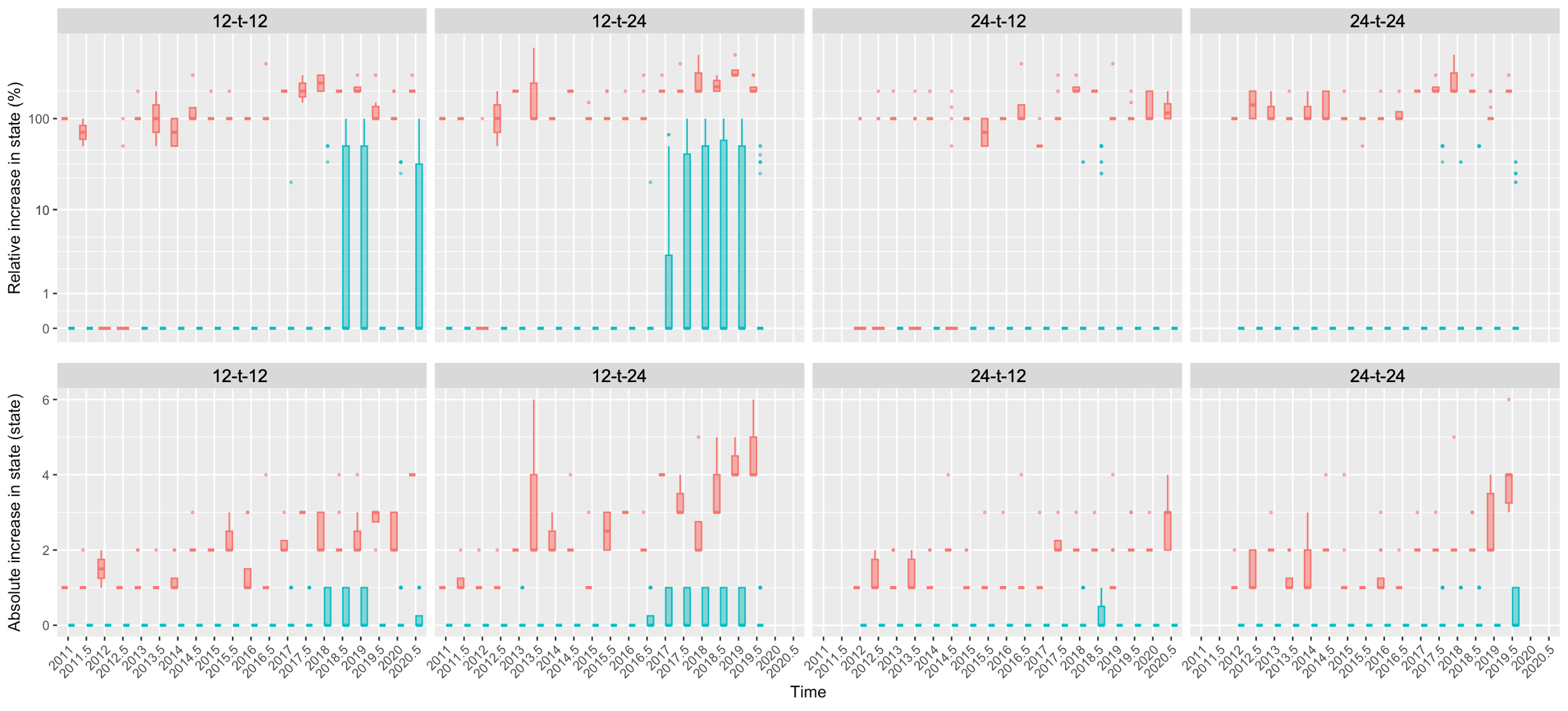


**Supplementary Figure S14.** Differences between success (>95^th^ percentile of each success measure: red) and unsuccess (<75^th^ percentile of each success measure: blue) of relative and absolute increases in number of states.


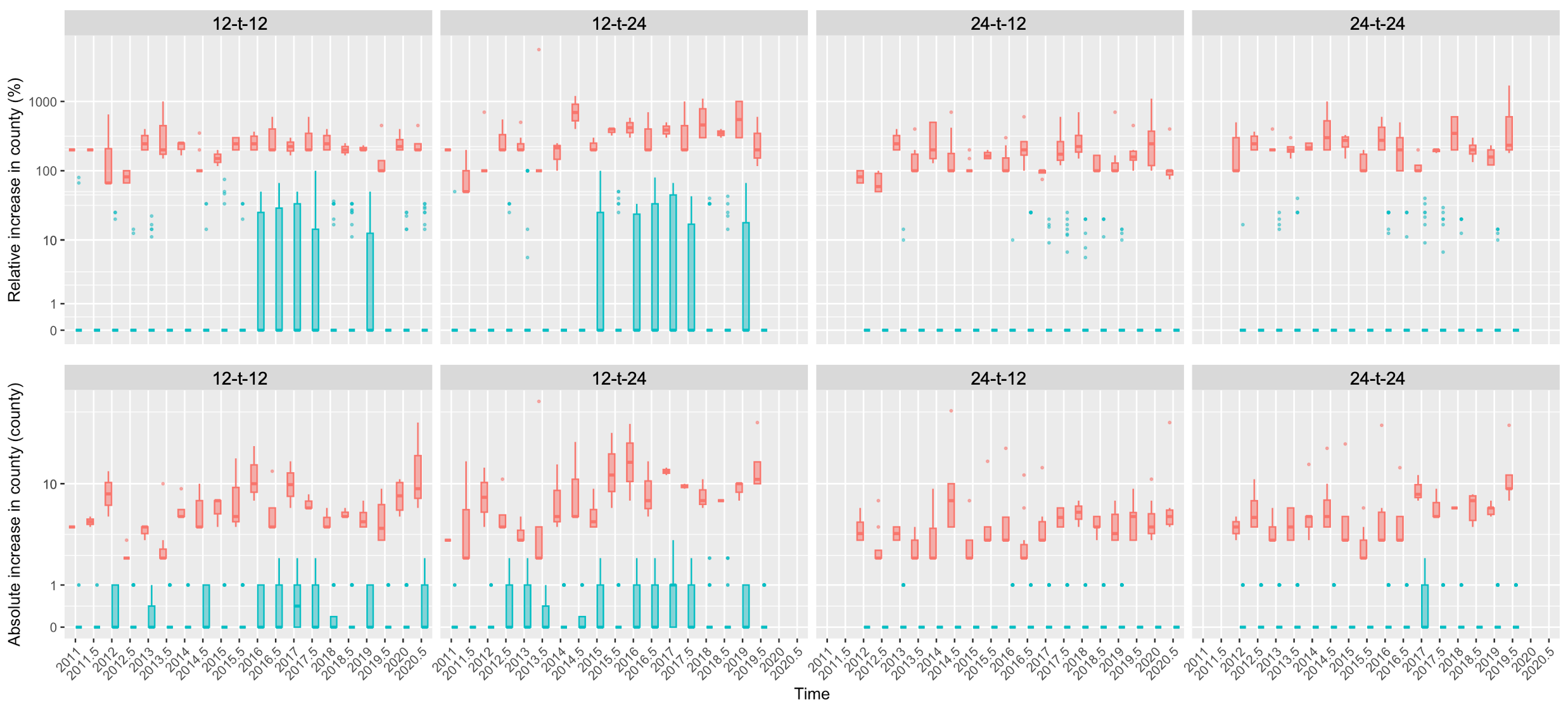


**Supplementary Figure S15.** Differences between success (>95^th^ percentile of each success measure: red) and unsuccess (<75^th^ percentile of each success measure: blue) of relative and absolute increases in number of counties.


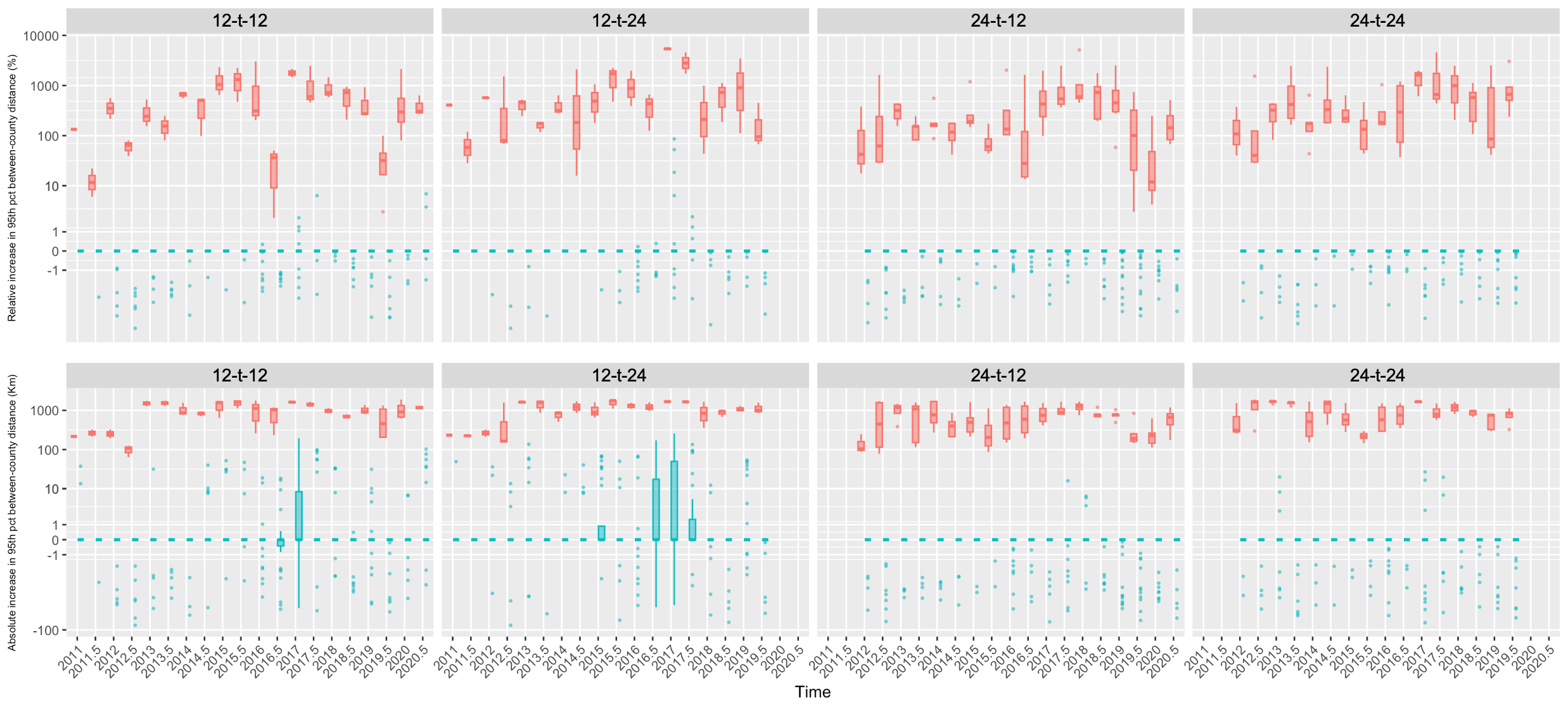


**Supplementary Figure S16.** Differences between success (>95^th^ percentile of each success measure: red) and unsuccess (<75^th^ percentile of each success measure: blue) of relative and absolute increases in 95^th^ percentile between-county distance.


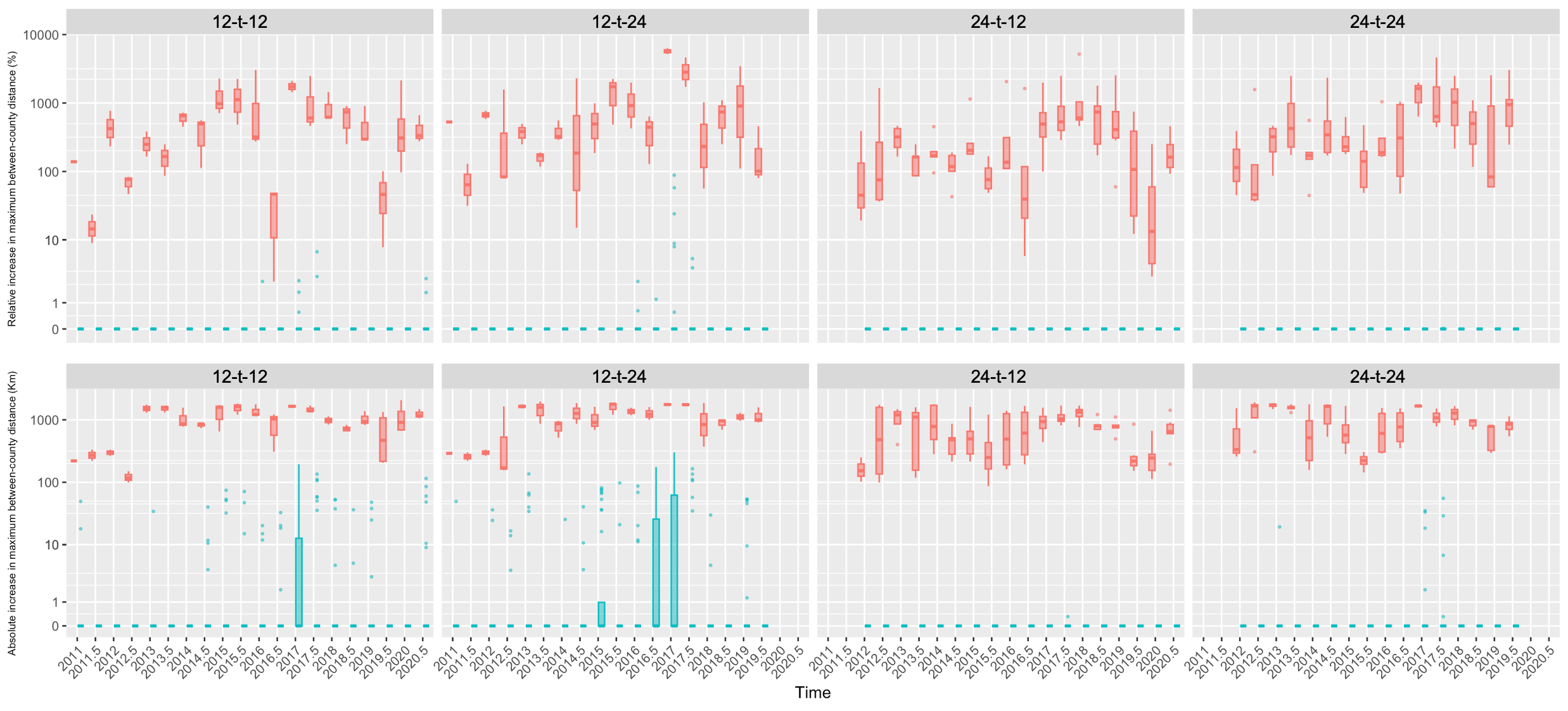


**Supplementary Figure S17.** Differences between success (>95^th^ percentile of each success measure: red) and unsuccess (<75^th^ percentile of each success measure: blue) of relative and absolute increases in maximum between-county distance.


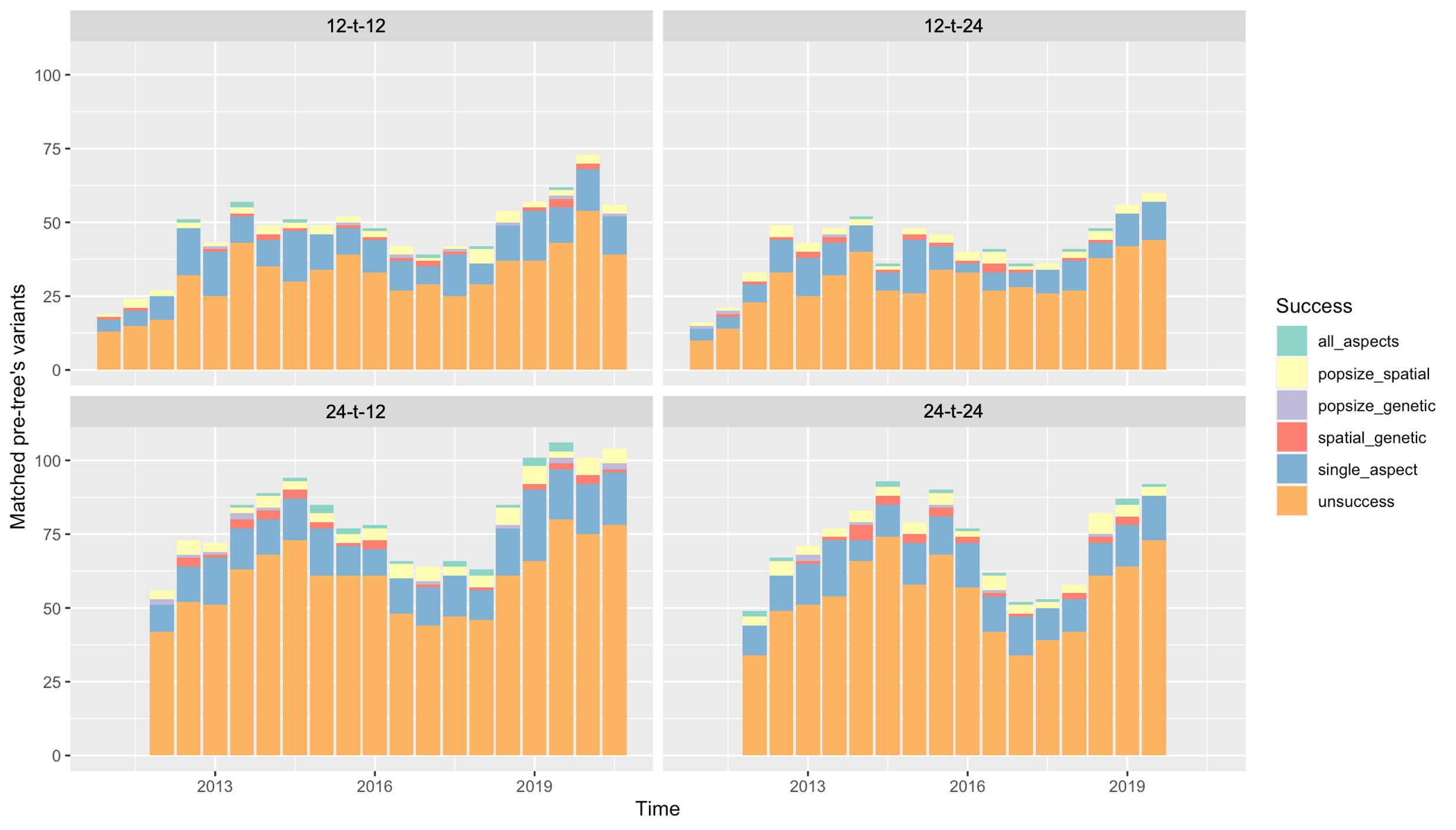


**Supplementary Figure S18.** Number of matched pre-tree’s variants that were not or were successful in one or more than one aspect (not including success in relative increase in number of states).


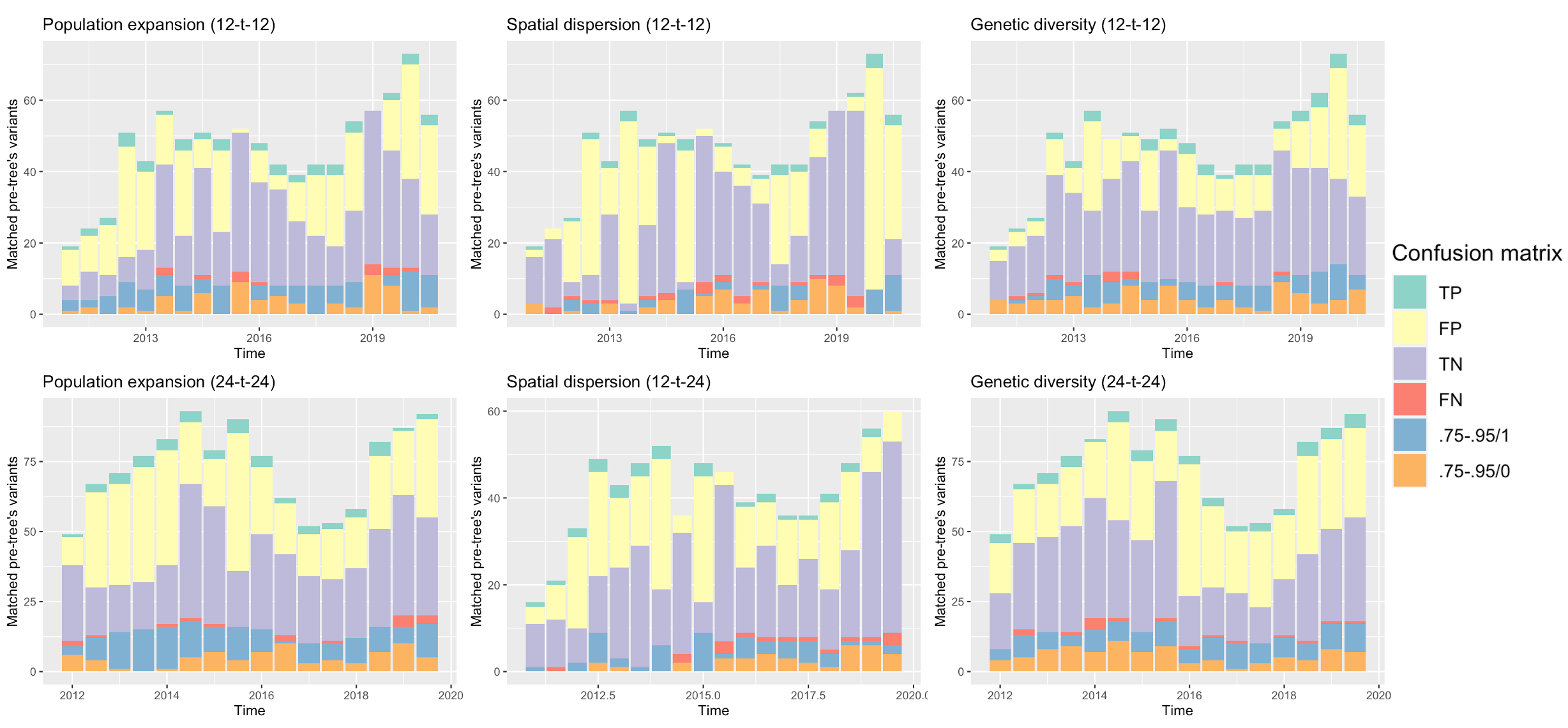


**Supplementary Figure S19.** Predictive performance of the best fit model of each success aspect and predicted period on the full dataset (2011-2020) demonstrated by confusion matrix components (TP: True positive, FP: False positive, TN: True negative, FN: False negative, .75-.95/1: Intermediate between success and unsuccess that was predicted to be positive, .75-.95/0: Intermediate between success and unsuccess that was predicted to be negative).


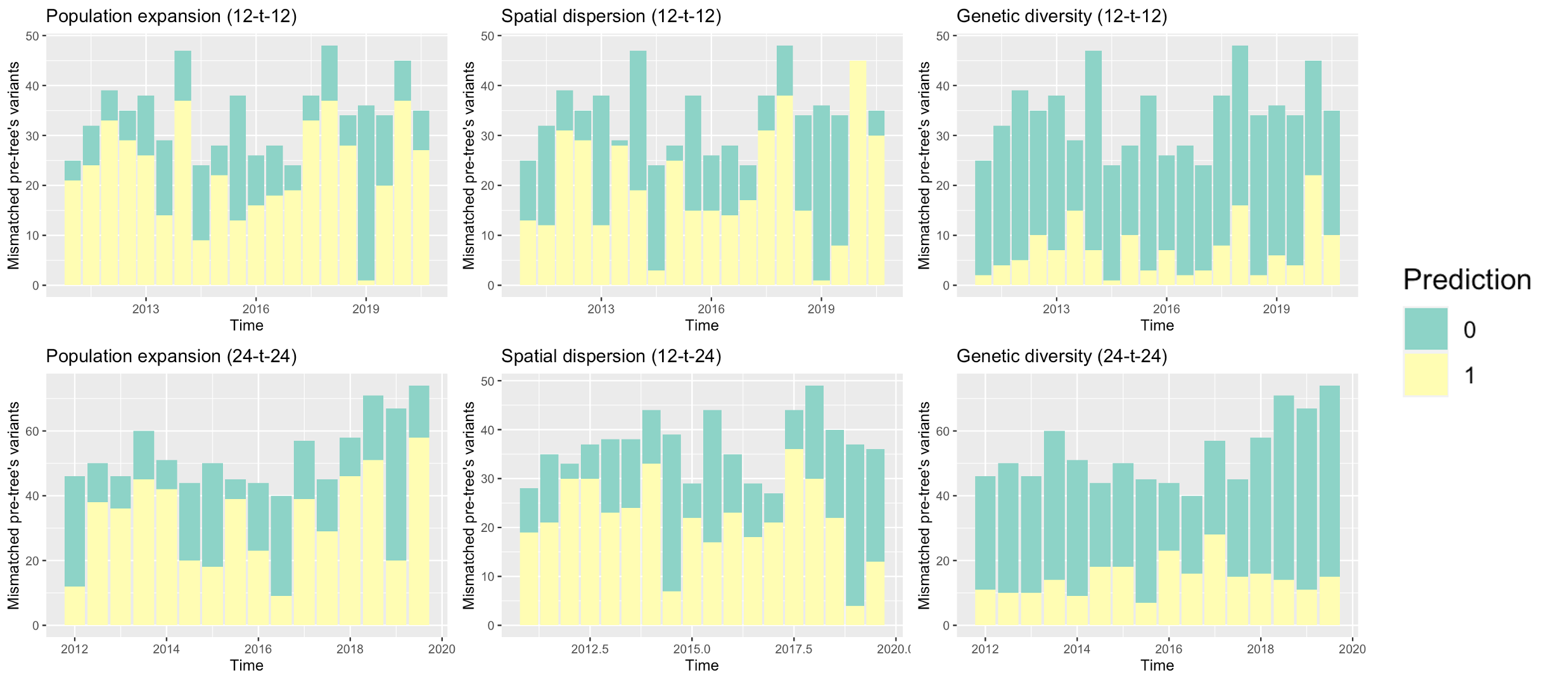


**Supplementary Figure S20.** Number of positive (1: yellow) and negative (0: turquoise) predicted mismatched pre-tree’s variants that the true success could not be measured.

**Supplementary Table S1** Predictive performance of all the models that were significantly better than null model based on likelihood ratio test (p-value < 0.05).

| **Success**  **aspect** | **Measure of**  **success** | **Scenario** | **Predictor** | | | | | | **Model**  **p-value (LRT)** | **Concordance** | **F1 score** | **Balanced**  **accuracy** |
| --- | --- | --- | --- | --- | --- | --- | --- | --- | --- | --- | --- | --- |
|  |  |  | **Branch**  **length** | **Ancestral**  **rate** | **Average**  **clade rate** | **LBI** | **Amino acid**  **distance** | **N-gly**  **Similarity** |  |  |  |  |
| Population  expansion | AbIn.Taxa | 24-*t*-12 | -41838.86 | 8519.93 | NA | 214.50 | 770.45 | 74.22 | 0.00 | 1.00 | NaN | 0.29 |
|  | AbIn.Taxa | 24-*t*-12 | -2694.61 | NA | -179.76 | 19.29 | 89.64 | 7.97 | 0.00 | 0.98 | 0.20 | 0.42 |
|  | AbIn.Taxa | 12-*t*-12 | -142.99 | NA | -200.03 | 8.11 | 20.88 | 1.40 | 0.00 | 0.88 | 0.50 | 0.67 |
|  | AbIn.Taxa | 12-*t*-12 | -157.87 | 291.50 | NA | 8.58 | 19.00 | 0.69 | 0.00 | 0.83 | 0.43 | 0.58 |
|  | ReIn.Taxa | 24-*t*-24 | 56.11 | 949.94 | NA | 25.55 | 17.33 | -2.50 | 0.00 | 0.86 | 0.57 | 0.75 |
|  | AbIn.Taxa | 24-*t*-24 | -165.95 | NA | -523.61 | 8.99 | 9.27 | 2.04 | 0.00 | 0.86 | 0.50 | 0.67 |
|  | AbIn.Taxa | 12-*t*-24 | -112.24 | -108.50 | NA | 5.25 | 13.47 | -1.90 | 0.00 | 0.83 | NA | 0.50 |
|  | AbIn.Taxa | 12-*t*-24 | -111.71 | NA | 44.46 | 5.14 | 13.20 | -2.32 | 0.01 | 0.83 | NA | 0.50 |
|  | AbIn.Taxa | 24-*t*-24 | -220.45 | 268.92 | NA | 8.89 | 7.72 | 0.20 | 0.00 | 0.81 | 0.57 | 0.75 |
|  | ReIn.Taxa | 24-*t*-24 | 32.70 | NA | 124.01 | 14.76 | 12.71 | 2.64 | 0.01 | 0.79 | 0.50 | 0.67 |
| Spatial  distribution | AbIn.State | 24-*t*-12 | -60.46 | 708.86 | NA | 18.05 | 47.59 | 9.27 | 0.00 | 0.93 | NA | 0.50 |
|  | AbIn.State | 24-*t*-12 | -31.38 | NA | 4683.94 | 29.29 | 114.23 | 14.60 | 0.00 | 0.93 | NA | 0.50 |
|  | AbIn.County | 24-*t*-12 | -163.81 | NA | -225.75 | 8.03 | -12.69 | 1.98 | 0.01 | 0.83 | 0.29 | 0.54 |
|  | AbIn.95County.Dist | 24-*t*-12 | -12.63 | NA | 435.43 | 8.18 | 3.04 | 3.86 | 0.02 | 0.81 | NA | 0.50 |
|  | ReIn.County | 24-*t*-12 | 27.25 | NA | 124.92 | 12.54 | -38.42 | -4.08 | 0.01 | 0.79 | 0.22 | 0.46 |
|  | AbIn.County | 24-*t*-12 | -161.63 | 61.23 | NA | 6.98 | -14.37 | 1.68 | 0.01 | 0.79 | 0.29 | 0.54 |
|  | ReIn.County | 24-*t*-12 | 25.75 | 284.62 | NA | 13.34 | -39.57 | -4.67 | 0.01 | 0.76 | 0.22 | 0.46 |
|  | AbIn.95County.Dist | 24-*t*-12 | -11.36 | 172.96 | NA | 8.92 | -0.36 | 3.46 | 0.02 | 0.76 | 0.46 | 0.63 |
|  | AbIn.State | 12-*t*-12 | -153.17 | 206.30 | NA | -4.85 | -9.55 | -0.36 | 0.05 | 0.73 | 0.22 | 0.46 |
|  | ReIn.MaxCounty.Dist | 12-*t*-12 | 19.87 | -898.66 | NA | 5.85 | 5.86 | 2.82 | 0.01 | 0.73 | 0.43 | 0.58 |
|  | ReIn.95County.Dist | 12-*t*-24 | -115.84 | NA | 1676.85 | 10.54 | 52.40 | 0.30 | 0.02 | 0.81 | 0.40 | 0.50 |
|  | ReIn.MaxCounty.Dist | 12-*t*-24 | -170.69 | -309.39 | NA | 5.51 | 53.43 | 0.79 | 0.03 | 0.79 | 0.50 | 0.67 |
|  | AbIn.State | 12-*t*-24 | 13.75 | NA | 519.81 | 6.20 | 29.98 | 1.62 | 0.04 | 0.77 | 0.44 | 0.58 |
|  | ReIn.95County.Dist | 12-*t*-24 | -114.13 | -245.62 | NA | 5.13 | 40.83 | 0.30 | 0.05 | 0.75 | 0.67 | 0.75 |
|  | ReIn.95County.Dist | 24-*t*-24 | 43.53 | NA | 1105.25 | 6.73 | 28.92 | 2.13 | 0.03 | 0.74 | 0.40 | 0.50 |
|  | AbIn.County | 12-*t*-24 | -28.60 | NA | 971.36 | 6.07 | -5.44 | -2.31 | 0.04 | 0.71 | 0.40 | 0.50 |
| Genetic  diversity | ReIn.95Nt.Dist | 12-*t*-12 | 100.11 | NA | 702.71 | 5.25 | -23.63 | -0.09 | 0.01 | 0.92 | 0.44 | 0.58 |
|  | AbIn.95Nt.Dist | 12-*t*-12 | 108.07 | -141.59 | NA | -1.22 | 2.07 | 0.56 | 0.00 | 0.92 | 0.62 | 0.79 |
|  | AbIn.95Nt.Dist | 12-*t*-12 | 110.38 | NA | 388.05 | 0.36 | 0.69 | 0.69 | 0.00 | 0.92 | 0.53 | 0.71 |
|  | ReIn.95Nt.Dist | 12-*t*-12 | 77.90 | -115.74 | NA | 1.98 | -15.82 | -0.11 | 0.03 | 0.88 | 0.62 | 0.79 |
|  | AbIn.95Nt.Dist | 24-*t*-12 | 59.92 | NA | -268.23 | 1.89 | 30.12 | -1.58 | 0.04 | 0.86 | 0.80 | 0.92 |
|  | AbIn.95Nt.Dist | 24-*t*-12 | 61.03 | -675.31 | NA | 1.19 | 41.41 | -1.07 | 0.01 | 0.83 | 0.55 | 0.71 |
|  | ReIn.95Nt.Dist | 24-*t*-12 | 88.33 | -458.75 | NA | 6.01 | -3.23 | -0.10 | 0.04 | 0.81 | 0.36 | 0.54 |
|  | ReIn.95Nt.Dist | 24-*t*-12 | 75.02 | NA | -1499.22 | 3.75 | -1.11 | -0.56 | 0.03 | 0.79 | 0.25 | 0.50 |
|  | ReIn.95Nt.Dist | 12-*t*-24 | 113.35 | -1846.66 | NA | -3.33 | 6.98 | 9.52 | 0.00 | 0.96 | NA | 0.50 |
|  | ReIn.95Nt.Dist | 12-*t*-24 | 110.75 | NA | -4324.44 | -4.79 | 9.56 | 8.14 | 0.00 | 0.94 | NA | 0.50 |
|  | AbIn.95Nt.Dist | 24-*t*-24 | 62.12 | -46.57 | NA | -0.19 | 42.52 | 1.77 | 0.01 | 0.86 | 0.50 | 0.67 |
|  | AbIn.95Nt.Dist | 24-*t*-24 | 75.09 | NA | -641.70 | 0.37 | 39.77 | 1.27 | 0.00 | 0.81 | 0.67 | 0.83 |
|  | ReIn.95Nt.Dist | 24-*t*-24 | -8.21 | -211.46 | NA | 3.07 | 58.97 | 4.12 | 0.02 | 0.79 | 0.50 | 0.67 |
|  | AbIn.95Nt.Dist | 12-*t*-24 | 66.55 | -411.01 | NA | -3.24 | -3.24 | 1.98 | 0.03 | 0.77 | 0.33 | 0.50 |
|  | ReIn.95Nt.Dist | 24-*t*-24 | -9.74 | NA | -596.74 | 3.11 | 62.10 | 4.30 | 0.02 | 0.76 | 0.40 | 0.58 |

**Supplementary Table S2** Predictive performance of the best fit model of each success aspect and predicted period on the full dataset (2011-2020).

| **Success aspect** | **Scenario** | **PPV*** | **NPV**** | **Sensitivity** | **F1 score** | **Specificity** | **Balanced accuracy** |
| --- | --- | --- | --- | --- | --- | --- | --- |
| Population expansion | 12-*t*-12 | 0.13 | 0.97 | 0.78 | 0.22 | 0.56 | 0.67 |
|  | 24-*t*-24 | 0.1 | 0.97 | 0.76 | 0.18 | 0.52 | 0.64 |
| Spatial distribution | 12-*t*-12 | 0.09 | 0.95 | 0.58 | 0.15 | 0.54 | 0.56 |
|  | 12-*t*-24 | 0.1 | 0.96 | 0.65 | 0.18 | 0.58 | 0.62 |
| Genetic Diversity | 12-*t*-12 | 0.16 | 0.98 | 0.81 | 0.27 | 0.66 | 0.74 |
|  | 24-*t*-24 | 0.11 | 0.97 | 0.77 | 0.19 | 0.52 | 0.65 |

*Positive predictive value

**Negative predictive value
